# π‐Extended Pleiadienes by [5+2] Annulation of 1‐Boraphenalenes and *ortho*‐Dihaloarenes

**DOI:** 10.1002/chem.202202053

**Published:** 2022-08-31

**Authors:** Matthias Schnitzlein, Chongwei Zhu, Kazutaka Shoyama, Frank Würthner

**Affiliations:** ^1^ Institut für Organische Chemie and Center for Nanosystems Chemistry (CNC) Universität Würzburg Am Hubland 97074 Würzburg Germany; ^2^ Key Laboratory of Functional Molecular Solids Ministry of Education and School of Chemistry and Materials Science Anhui Normal University Wuhu 241002 P. R. China

**Keywords:** annulation, aromaticity, azulene, cyclodehydrogenation, polycyclic aromatic hydrocarbons

## Abstract

Palladium‐catalyzed [5+2] annulation of 1‐boraphenalenes with *ortho*‐dihaloarenes afforded negatively curved π‐extended pleiadienes. Two benzo[1,2‐*i*:4,5‐*i’*]dipleiadienes (BDPs) featuring a seven‐six‐seven‐membered ring arrangement were synthesized and investigated. Their crystal structure revealed a unique packing arrangement and theoretical calculations were employed to shed light onto the dynamic behavior of the BDP moiety and its aromaticity. Further, a naphthalene‐fused pleiadiene was stitched together by oxidative cyclodehydrogenation to yield an additional five‐membered ring. This formal azulene moiety led to distinct changes in optical and redox properties and increased perturbation of the aromatic system.

## Graphical abstract

**Intrigued by their (anti)aromaticity** and expected negative curvature, a series of π‐extended pleiadienes has been synthesized by palladium‐catalyzed [5+2] annulation. Their structural, redox and optical properties were probed and further investigated by theoretical calculations.

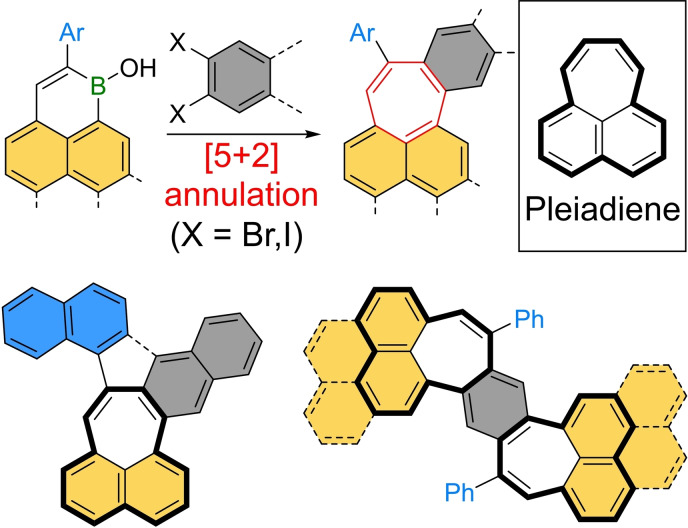

## Introduction

**Scheme 1:**
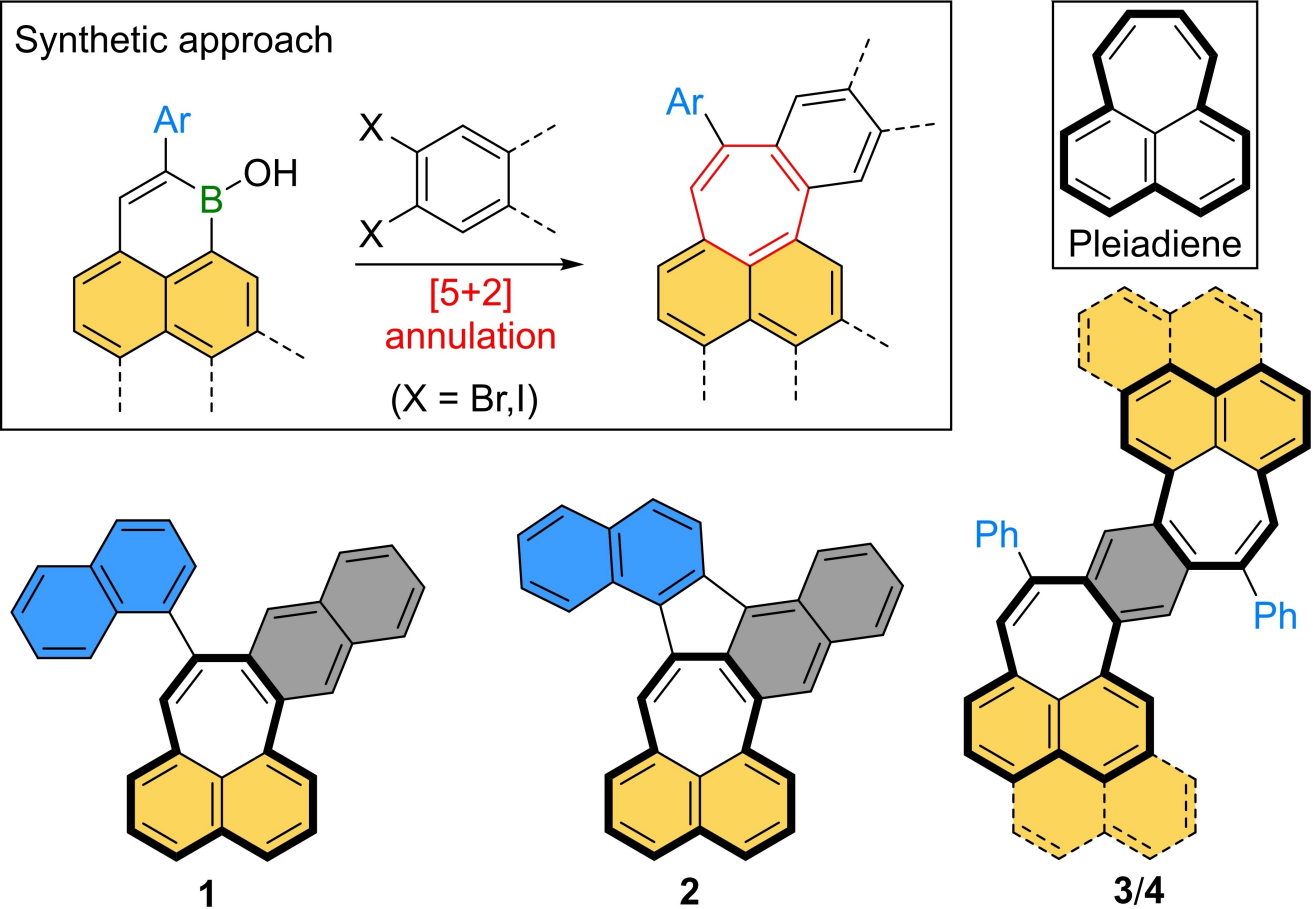
Synthetic approach towards seven‐membered ring buildup by [5+2] annulation with chemical structures of the new molecules **1**–**4** presented herein.

Pleiadiene – cyclohepta[*de*]naphthalene (Scheme 1 top right) – represents one of the most fundamental heptagon‐containing polycyclic aromatic hydrocarbons (PAHs). It was first mentioned in 1933^[1]^ and its close relative acepleiadiene was first synthesized in 1951.^[2]^ The synthesis of pristine pleiadiene was finally reported in 1956.^[3]^ Initially hypothesized to show similar aromaticity to anthracene and phenanthrene, Boekelheide and Vick reasoned that Hückel's rule could not be justified for *peri*‐fused PAHs, prompted by their experimental results such as the readily undergone Diels‐Alder reaction of pleiadiene with maleic anhydride.^[3]^ Apart from their intriguing anti‐ or non‐aromaticity, such non‐alternant PAHs also exhibit compelling structural, optoelectronic and magnetic properties.^[4]^ Particularly through further embedment of the pleiadiene heptagon into a larger carbon scaffold, negative curvature and thus a saddle‐shape can be obtained. This was first shown in 1988 through the synthesis and structural elucidation of [7]circulene.^[5]^ Since then synthetic progress was limited, presumably due to a lack of synthetic strategies for the construction of seven‐membered ring systems. As such, the last decade saw a growing number of studies on the synthesis of these carbon scaffolds. Common strategies include intramolecular cyclodehydrogenation,^[6]^ Friedel‐Crafts acylation,^[7]^ π‐expansion of cycloheptanones,^[8]^ ring expansion of cyclohexanones^[9]^ and HF elimination of aryl fluorides on activated alumina,^[10]^ and thus require special precursors. Transition metal‐catalyzed C−C bond formation reactions offer greater flexibility through a broader substrate scope, but to date only few examples have been published including palladium‐catalyzed intramolecular C−H arylation,^[11]^ [3+2+2],^[12]^ [4+3]^[13]^ and [5+2] annulation reactions,^[14]^ as well as cobalt‐catalyzed cyclotrimerization.^[15]^ Recently, our group has reported a method for the construction of heptagon‐embedded PAHs by palladium‐catalyzed [5+2] annulation of cyclic borinic acids with aryl halides (Scheme 1 top left).^[16]^ Since then we have used this strategy to synthesize PAHs with two embedded azulene units,^[17]^ as well as PAHs with both negative and positive curvature.^[18]^ We conjectured that by using 1‐boraphenalenes as the borinic acid precursor for this synthetic method a series of pleiadiene derivatives could be synthesized.

Herein, we present the synthesis of a series of π‐extended pleiadienes (**1** and **2**) and benzo[1,2‐*i*:4,5‐*i*’]dipleiadienes (BDPs, **3** and **4**) and investigate their aromaticity. The seven‐membered rings were constructed by [5+2] annulation of 1‐boraphenalenes and *ortho*‐dihaloarenes. A formal azulene moiety could be generated by a following oxidative cyclodehydrogenation, wherein the orthogonal naphthalene moiety of **1** was fused to its seven‐membered ring. Investigations of these molecules’ aromatic character revealed significantly lower antiaromaticity of the heptagonal ring compared to pristine pleiadiene and a correlation between its antiaromaticity and the oxidation potentials.

## Results and Discussion

### Synthesis

**Scheme 2:**
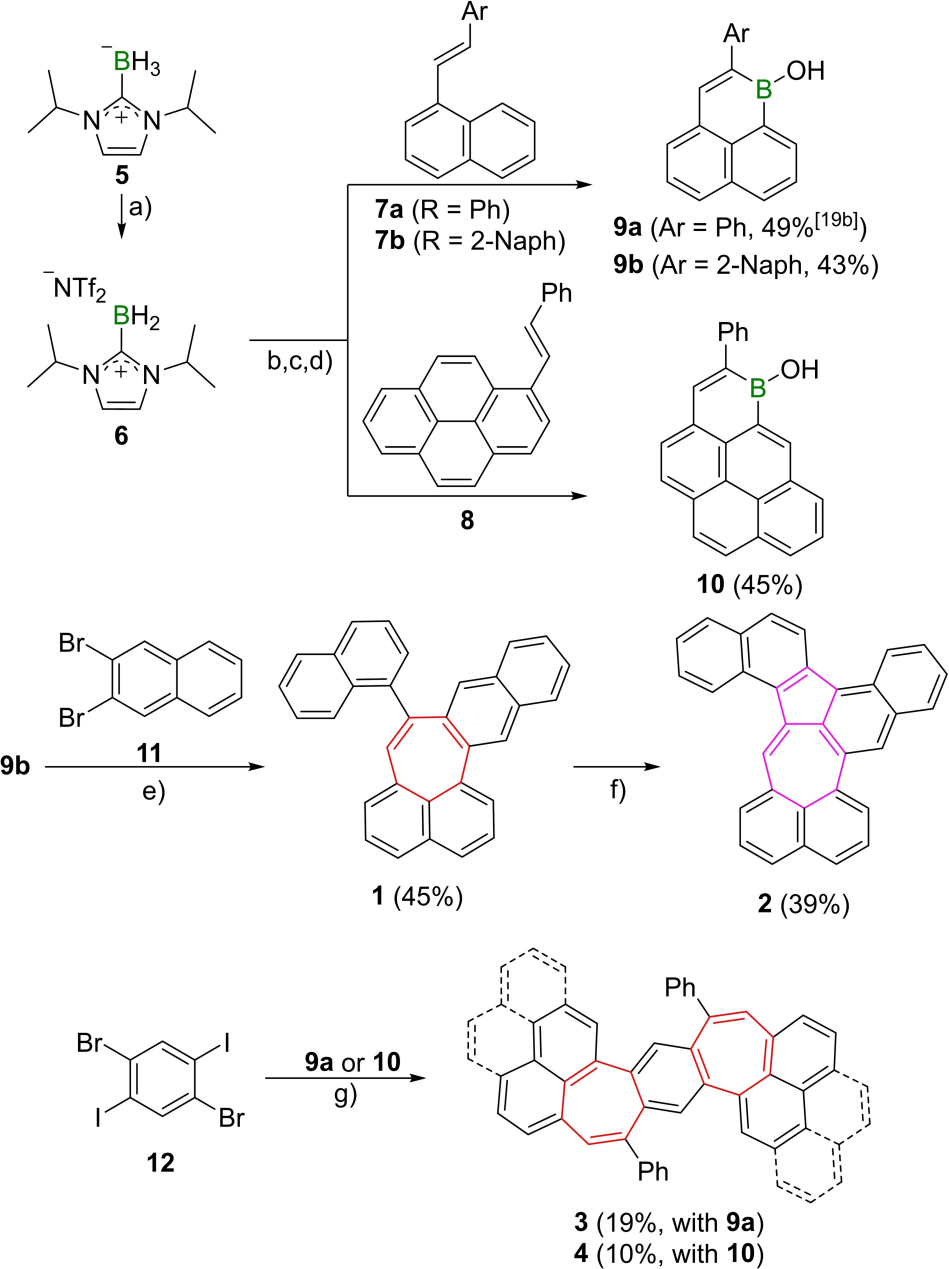
Synthesis of **1**–**4** by [5+2] annulation. a) 1.2 equiv. **5**, HNTf (1.22 equiv.), PhCl, rt, 90 min. b) alkene (1.0 equiv.), PhCl, 110 °C, 5 h. c) TEMPO (2.3 equiv.), PhCl, 80 °C, 36 h. d) hydrolysis (yield over four steps). e) **11** (2.0 equiv.), [Pd_2_(dba)_3_] ⋅ CHCl_3_ (1.5 mol %), P(^*t*^Bu)_3_ ⋅ HBF_4_ (3.6 mol %), Cs_2_CO_3_ (3.3 equiv.), H_2_O (10 equiv.), ^*t*^AmOH, 100 °C, 42 h. f) DDQ (1.0 equiv.), TfOH, CH_2_Cl_2_, 0 °C, 15 min. g) borinic acid (2.4 equiv.), [Pd_2_(dba)_3_] ⋅ CHCl_3_ (6 mol %), P(^*t*^Bu)_3_ ⋅ HBF_4_ (14 mol %), Cs_2_CO_3_ (6.6 equiv.), H_2_O (20 equiv.), ^*t*^AmOH, 100 °C, 42 h. Tf: trifluoromethanesulfonyl, TEMPO: 2,2,6,6‐tetramethylpiperidinyloxyl, dba: dibenzylideneacetone, DDQ=2,3‐dichloro‐5,6‐dicyano‐1,4‐benzoquinone.

We first describe the synthesis of the borinic acid precursors **9 a**/**b** and **10** through our one‐pot C−H borylation protocol (Scheme 2).^[19]^ We started synthesis from NHC‐borane complex **5**, which was transformed to its corresponding borenium cation **6** under liberation of hydrogen gas by treatment with the Brønsted superacid bistriflimidic acid (HNTf_2_).^[19]^ This reactive species then facilitated hydroboration and electrophilic borylation of alkenes **7 a**/**b** and **8**. Following TEMPO‐mediated dehydrogenation and hydrolysis produced the borinic acids **9 a**/**b** and **10** in good yields of 43–49 %.^[19]^ Borinic acid **9 b** underwent annulation with 2,3‐dibromonaphthalene (**11**) to give **1** in a moderate yield of 45 %. Subsequent oxidative dehydrogenation with one equivalent of 2,3‐dichloro‐5,6‐dicyano‐1,4‐benzoquinone (DDQ) in triflic acid/dichloromethane afforded **2** with a formal azulene moiety in 39 % yield. For the benzodipleiadienes the seven‐membered ring formation of 1,2,4,5‐tetrabromobenzene with **9 a** lead to an inseparable mixture of two regioisomers due to the inherent *C*_1_ symmetry of the cyclic borinic acids (i. e. *C*_2_‐symmetric **3** and its *C*_s_‐symmetric counterpart with both phenyls on the same side). We therefore utilized 1,4‐diiodo‐2,5‐dibromobenzene (**12**) as halide coupling partner for **9 a** and **10**. Under standard [5+2] annulation reaction conditions BDPs **3** and **4** were obtained in 19 % and 10 % yield, respectively, without the formation of regioisomeric products. Whereas **1** exhibits excellent solubility in common organic solvents, **2** and **3** only possess limited solubility and **4** is only sparingly soluble, even in chlorinated solvents. All compounds are bench‐stable for extended periods of time as solids.

### Structural properties

**Figure 1:**
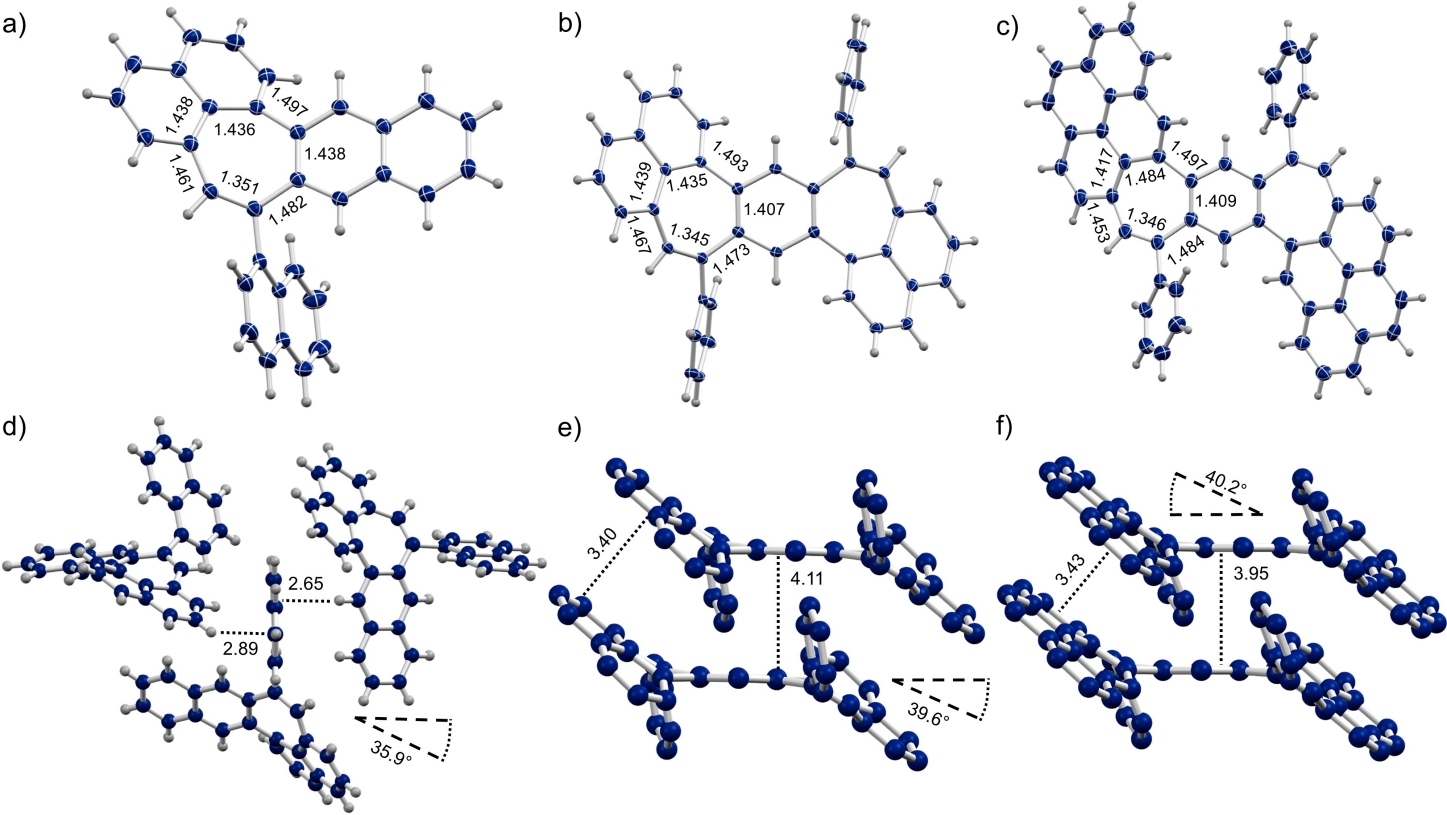
Solid state structures of molecules **1**, **3** and **4** (left to right). a–c) Crystal structure with ellipsoids set at 50 % probability. d–f) Solid state packing and select distances and angles. All lengths are given in Å, hydrogens omitted for clarity in e) and f).

We were able to grow single crystals of **1**, **3** and **4** suitable for X‐ray diffraction analysis and thus could assess their structure and solid‐state packing (Figure 1).^[20]^ All three compounds exhibit comparable bond lengths in their seven‐membered rings each featuring a shorter bond with a length between 1.345 Å and 1.351 Å between the heptagon C−H and the orthogonal phenyl/naphthyl groups. These bond lengths are in line with the values usually obtained for conjugated double bonds like in styrene (−C=C−, 1.339 Å)^[21]^ and hint at a pronounced olefinic character of these bonds. This is further corroborated by the chemical shifts of the corresponding heptagon proton in their ^1^H NMR spectra, where the proton signal is shifted significantly upfield to values between 6.70 ppm and 6.97 ppm, indicating shielding. For **1** the other six bonds in the seven‐membered ring have bond lengths ranging from 1.436 Å to 1.497 Å and thus are significantly longer than aromatic C−C bonds (−C_ar_≃C_ar_−, 1.397 Å)^[21]^ and comparable to single bonds between aromatic systems (−C_ar_−C_ar_−, 1.490 Å).^[21]^ For the BDP moieties however, the bond bordering on the central benzene ring exhibits a length of 1.407 Å for **3** and 1.409 Å for **4** and as such matches the bond length of aromatic C−C bonds. The five remaining bonds of their heptagon moiety are elongated compared to typical aromatic bond lengths and range between 1.417 and 1.497 Å. The seven‐membered ring and its saddle‐shape strongly tilts the fused naphthalene and pyrene moieties out of plane. The BDP core induces a stronger tilt with angles around 40° in **3** and **4**, whereas the single heptagon of **1** only amounts to an angle of 36°. Further, the benzo[1,2‐*i*:4,5‐*i*’]dipleiadiene core induces a slipped stack packing arrangement, in which π‐π interactions between the central benzene ring and the peripheral naphthalene/pyrene moieties are present (Figure 1d,f). The distance between the peripheral π‐system amounts to 3.40 and 3.43 Å for **3** and **4**, respectively. The distance between the central benzene rings measures 4.11 Å in the case of **3** and 3.95 Å in the case of **4**. Expectedly, the latter thus exhibits stronger π‐π interactions since it also features the larger π‐scaffold. These packing arrangements with their close stacking also explain the rather limited solubility of both BDP‐containing scaffolds. Conversely, **1** with its sole seven‐membered ring and great solubility features only very weak intermolecular interactions in the solid state. As such, only C−H‐π interactions between the orthogonal naphthyl groups and the cycloheptanaphthalene moieties were observed with distances of 2.65 Å and 2.89 Å, respectively.

**Figure 2:**
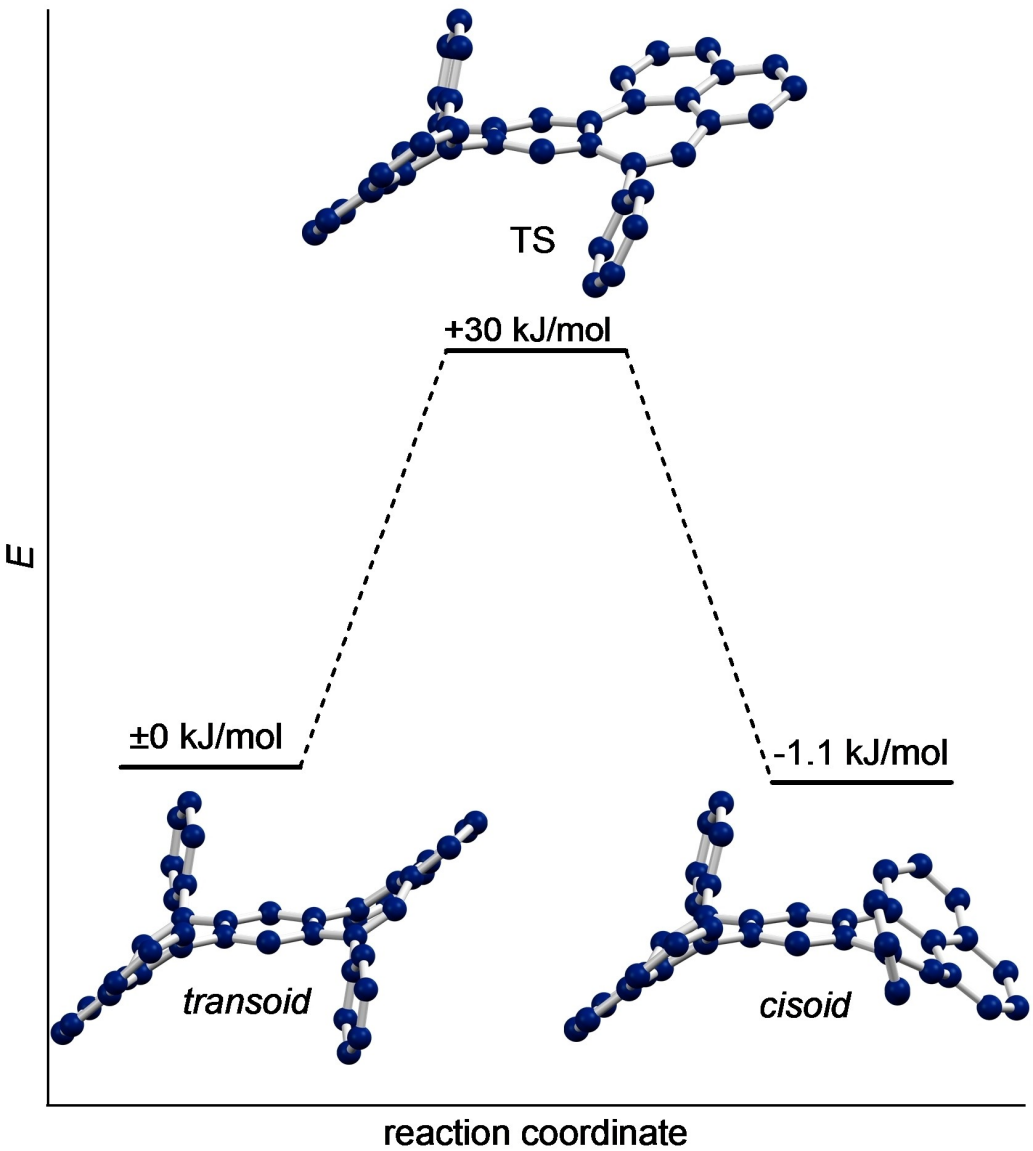
Interconversion between the *transoid* and *cisoid* conformers of **3** by inversion of the seven‐membered ring as calculated at the B3LYP/6‐31+G(d) level of theory.

In order to obtain insight into the conformational properties of **3** and **4** we performed theoretical calculations. Both compounds exclusively exhibit a *transoid*, S‐shape in their crystal structure (Figure 1e, f). Through inversion of one of the seven‐membered rings a *cisoid*, C‐shaped conformation can be obtained. Calculations of **3** at the B3LYP/6‐31+G(d) level of theory put both confirmations at the same energy with a miniscule difference of only 1.1 kJ/mol. Further, a transition state with one planarized seven‐membered ring and a very low barrier of only 30 kJ/mol was obtained computationally (Figure 2).

### Electrochemical and optical properties

We then conducted electrochemical measurements to assess the oxidation potentials of the synthesized compounds (Table 1). The parent compound to these heptagon‐embedded PAHs, cycloheptatrienyl (C_7_H_7_), is markedly non‐aromatic, but obtains aromaticity by one‐electron oxidation to the 6π tropylium cation, satisfying Hückel's [4*n*+2] rule. In accordance with such a gain in aromaticity, compounds **1** and **2** exhibit one rather low‐lying oxidation potential. The decrease in oxidation potential is typical for PAHs with formal azulene moieties compared to their seven‐membered ring containing analogues.[^16^, ^17^] The BDP‐type compounds **3** and **4** feature two oxidation processes at even lower potentials of +0.57 V/+0.89 V and +0.44 V/+0.69 V, respectively. The lower oxidation potential of **4** than that of **3** might be attributed to a more extended π‐system of **4**.

**Table 1:** Optical and oxidation properties of **1–4**.

	*λ*_abs_[nm] (*ϵ* [L mol^−1^ cm^−1^])^[a]^	*λ*_em_ [nm]^[b]^ (*Φ*_em_)^[c]^	*E*_ox_ [V]^[d]^
**1**	378 (8900), 300 (17400)	474 (0.23)	+0.72
**2**	441 (14100), 320 (24900), 277 (49300)	n.d.	+0.55
**3**	402 (20600), 328 (33000), 298 (32400)	558 (0.07)	+0.57, +0.89
**4**	437 (20500), 345 (26100), 302 (49900)	624 (0.02)	+0.47, +0.69

[a] UV/Vis absorption spectra were measured in dichloromethane (*c*≈1×10^−5^ m) at 298 K. [b] Fluorescence spectra were measured in dichloromethane (*c*≈1×10^−7^ m) at 298 K with the highest wavelength absorption maxima as excitation wavelength. [c] Absolute fluorescence quantum yields were measured in dichloromethane (*c*≈1×10^−5^ m) 298 K and are uncorrected. [d] CV and SW/DPV spectra were measured at room temperature in dry, degassed dichloromethane (*c*≈2.5×10^−4^ m) with 0.1 m (*n*‐Bu)_4_NPF_6_ under an argon atmosphere and are calibrated to the ferrocene/ferrocenium redox couple. n.d.=not detected.

Optical measurements revealed strong ultraviolet absorption of compounds **1**–**4** with extinction coefficients between 17400 and 49300 L mol^−1^ cm^−1^ (Table 1, Supporting Information Figures S13–16). PAHs **1** and **3** exhibit weak absorption bands between 375 and 405 nm causing their yellow color. Compared to **1**, the second seven‐membered ring of the BDP moiety in **3** causes slightly higher wavelength absorption with significantly increased extinction coefficients. The absorption of **2** and **4** is further bathochromically shifted with maxima around 440 nm and extinction coefficients around the low 10^3^ L mol^−1^ cm^−1^ range responsible for their red color. Upon excitation **1** emits blue light (*λ*_em_=474 nm) with a quantum yield of 23 %, whereas the BDP moiety leads to higher wavelength emission in the yellow (*λ*_em_=558 nm) or red domain (*λ*_em_=624 nm) for **3** and **4**, respectively. Conversely, fluorescence is quenched for **2** in dichloromethane solutions presumably due to the energy loss of its excited state by flipping motion and intramolecular charge transfer as seen often for azulene‐containing PAHs. Both BDP‐embedded compounds **3** and **4** feature drastically reduced quantum yields compared to **1** which might originate from their biradical character and structural relaxations in the excited state.

### NICS and AICD analysis

**Figure 3:**
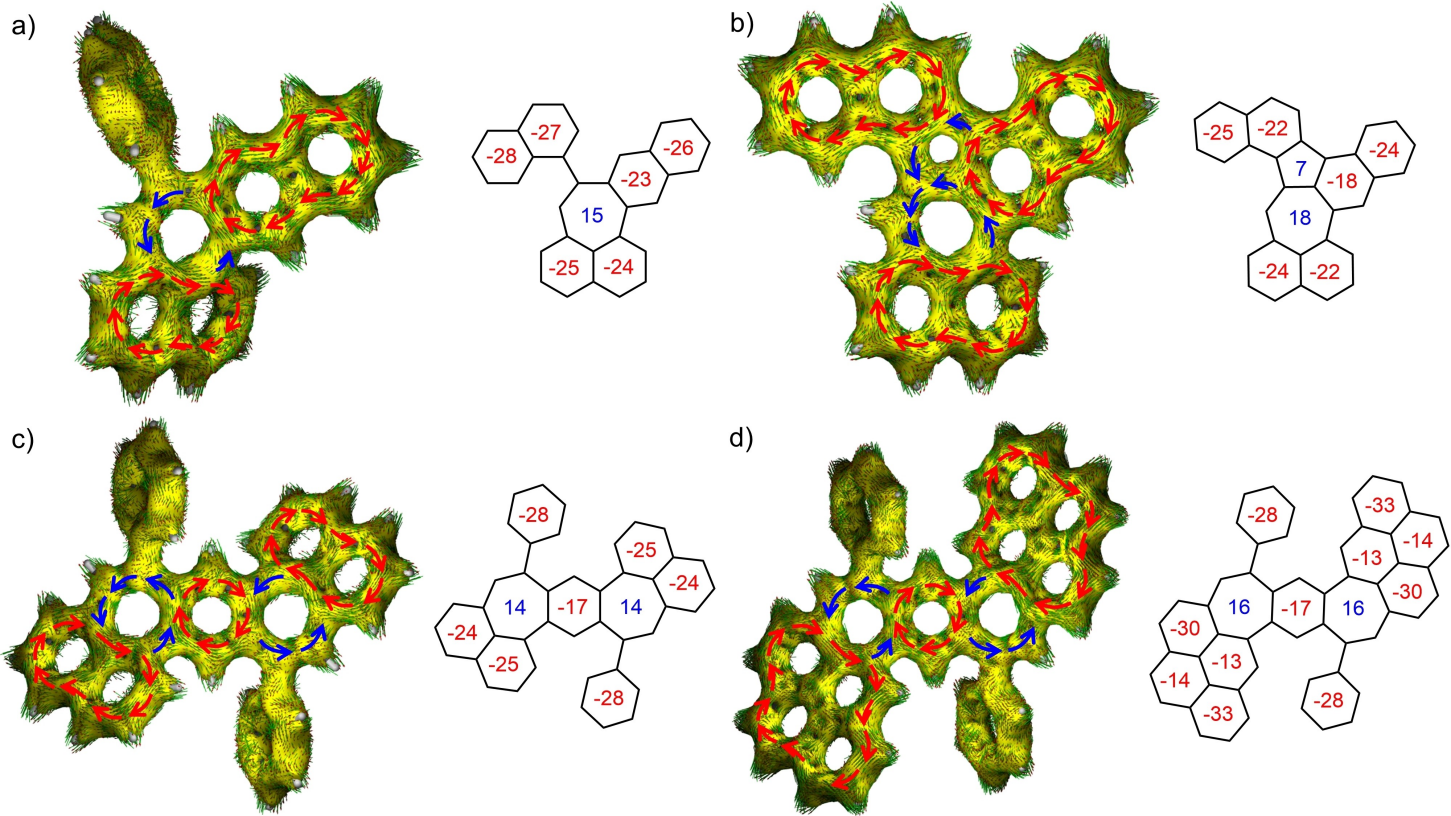
AICD plots and NICS(1)_zz_ values of molecules **1** (a), **2** (b), **3** (c) and **4** (d) calculated at the B3LYP/6‐31+G(d) level of theory with aromatic clock‐wise ring currents colored in red and anti‐aromatic, counterclockwise ring currents in blue.

Intrigued by their unique non‐benzenoid structures, we investigated the aromaticity of pleiadienes **1**–**4** by theoretical calculations. Therefore, nucleus‐independent chemical shifts (NICS)^[22]^ were calculated and anisotropy of the induced current density (AICD)^[23]^ plotted (Figure 3). Due to their non‐planar nature, the NICS_zz_ values one Ångstrom above and below the plane of reference differ and were averaged for an accurate assessment of NICS(1)_zz_ values (Supporting Information Table S4).^[24]^ As a baseline we also calculated the NICS(1)_zz_ values for pristine pleiadiene which amounted to values of −20 for both benzenoid rings and 30 for its *peri*‐fused seven‐membered ring (Supporting Information Table S4).

The BDP moiety of **3** and **4** is characterized by a central aromatic benzene ring with nucleus‐independent chemical shifts of −17 adjoined by antiaromatic seven‐membered rings with NICS(1)_zz_ values of 14 and 16, respectively, indicating rather strong antiaromaticity. Due to non‐planarity these values are smaller than that for pristine pleiadiene. Both values can also be correlated to their respective oxidation potentials with higher antiaromaticity also corresponding to more facile oxidation. Furthermore, the peripheral naphthalene and pyrene moieties retain their individual aromaticity. The pyrenes of **4** even display a strong difference between very aromatic rings with values of around −30 and weaker aromatic rings with around −13. This is akin to pristine pyrene, which displays a rather strong olefinic character in the two central benzene rings explained by its optimal distribution of Clar sextets. These findings are corroborated by the respective AICD plots. Similarly, the naphthalene moieties of **3** behave almost like individual naphthalene with strong aromaticity evidenced by NICS(1)_zz_ values of around −25, similar to the orthogonal phenyl groups with a shift of −28. As such, **3** and **4** can be regarded as naphthalenes and pyrenes slightly disturbed by fusing them onto the BDP scaffold with only localized aromaticity in its central benzene ring. Their frontier molecular orbitals, however, are located over the whole molecule excluding the orthogonal phenyl groups. Singly heptagon‐embedded **1** exhibits a similar phenomenon: The two naphthalene units fused to the seven‐membered ring remain strongly aromatic but interacting through the central heptagon with a NICS(1)_zz_ value of 15 as evidenced by delocalized frontier molecular orbitals over the whole molecule. Interestingly, the orthogonal naphthalene moieties also retain their individual aromaticity upon additional C−C bond formation, so that in **2** three quasi‐individually aromatic naphthalenes are present. They are then perturbed and interacting through a formal azulene moiety with a strongly anti‐aromatic seven‐membered and a weakly anti‐aromatic five‐membered ring with NICS(1)_zz_ values of 18 and 7, respectively. The naphthalenes also display aromatic ring currents in their AICD plots adjoined by anti‐aromatic, counterclockwise currents of the azulene. Thus, the non‐interacting orthogonal naphthalene unit of **1** is incorporated in the scaffold of **2** and increases the antiaromaticity of its heptagonal ring. This is most likely causing the notable bathochromic shift observed in the UV/Vis absorption and the increased susceptibility of the seven‐membered ring to oxidation.

## Conclusion

To summarize, we were able to extend our recently introduced [5+2] annulation protocol to furnish novel pleiadienes with either a benzo[1,2‐*i*:4,5‐*i*’]dipleiadiene (BDP) core or an azulene moiety. Through the BDP moiety a warped, S‐shape with a distortion of around 40° is imparted onto the scaffold as confirmed by single crystal X‐ray analysis. These *transoid* or S‐shaped conformers rapidly undergo conformational flipping of the heptagonal ring at room temperature towards the *cisoid*, C‐shaped conformers via an activation barrier of only 30 kJ/mol. Further, facile oxidation potentials are imparted through the seven‐membered rings that are in line with their anti‐aromaticity as described by their NICS(1)_zz_ values. The formation of the azulene moiety by oxidative cyclodehydrogenation leads to significant increase in absorbance and a bathochromical shift thereof in conjunction with fluorescence quenching and a decreased oxidation potential compared to its parent molecule with only a seven‐membered ring. Theoretical calculations revealed that all systems feature quasi‐independent aromatic moieties adjoined by anti‐aromatic seven‐ and five‐membered ring systems with delocalized frontier molecular orbitals.

## Experimental Section

**Synthesis of 1**: In a Schlenk tube equipped with a magnetic stirring bar were weighed in borinic acid **9 b** (31 mg, 0.10 mmol, 1.0 equiv.), 2,3‐dibromonaphthalene (57 mg, 0.20 mmol, 2.0 equiv.), [Pd_2_(dba)_3_] ⋅ CHCl_3_ (1.6 mg, 1.5 μmol, 1.5 mol %), P(^*t*^Bu)_3_ ⋅ HBF_4_ (1.0 mg, 3.6 μmol, 3.6 mol %) and cesium carbonate (0.11 g, 0.33 mmol, 3.3 equiv.). Under nitrogen atmosphere distilled water (18 μL, 1.0 mmol, 10 equiv.) and *tert*‐amyl alcohol (3 mL) were added by syringe. The mixture was stirred at room temperature for 1 h and then at 100 °C for 42 h. It was cooled to room temperature, filtered through celite and the filter cake was washed with ethyl acetate. After evaporation of the solvent from the filtrate the residue was purified by silica‐gel column and preparative thin‐layer chromatography (eluent *n‐*hexane:ethyl acetate 19 : 1 by volume) followed by washing with methanol.

**Synthesis of 2**: In a Schlenk tube equipped with a magnetic stirring bar were weighed in PAH **1** (12.0 mg, 29.7 μmol, 1.0 equiv.) and DDQ (6.73 mg, 29.7 μmol, 1.0 equiv.). Under nitrogen atmosphere the solids were dissolved in dry dichloromethane (15 mL). After cooling to 0 °C triflic acid (0.15 mL) was added, whereupon the reaction mixture turned black. After stirring for 15 min at 0 °C the reaction was quenched by addition of saturated aqueous sodium bicarbonate. The phases were separated, the aqueous phase extracted with dichloromethane (2×20 mL) and the combined organic phases washed with water (3×25 mL) before drying over magnesium sulfate. The crude was then purified by silica gel column chromatography (eluent cyclohexane:dichloromethane 4 : 1 by volume) and washed with methanol.

**General procedure for the synthesis of BDPs 3 and 4**: In a Schlenk tube equipped with a magnetic stirring bar were weighed in borinic acid **9 a** or **10** (2.4 equiv.), 1,4‐dibromo‐2,5‐diiodobenzene (1.0 equiv.), [Pd_2_(dba)_3_] ⋅ CHCl_3_ (6 mol %), P(^*t*^Bu)_3_ ⋅ HBF_4_ (14 mol %) and cesium carbonate (6.6 equiv.). Under nitrogen atmosphere distilled water (20 equiv.) and *tert*‐amyl alcohol were added by syringe. The mixture was stirred at room temperature for one hour and then at 100 °C for 42 h. After cooling to room temperature, the reaction mixture was filtered through celite and eluted with ethyl acetate and dichloromethane. After evaporation of the solvent from the filtrate the residue was purified by two‐time silica gel column chromatography (eluents *n‐*hexane:ethyl acetate and *n*‐hexane:dichloromethane).

## Conflict of interest

The authors declare no conflict of interest.

1

## Supporting information

As a service to our authors and readers, this journal provides supporting information supplied by the authors. Such materials are peer reviewed and may be re‐organized for online delivery, but are not copy‐edited or typeset. Technical support issues arising from supporting information (other than missing files) should be addressed to the authors.

Supporting Information

## Data Availability

The data that support the findings of this study are available in the supplementary material of this article.

## References

[1] F. Louis , M. Fieser , J. Am. Chem. Soc. 1933, 55, 3010–3018.

[2] V. Boekelheide , W. E. Langeland , C.-T. Liu , J. Am. Chem. Soc. 1951, 73, 2432–2435.

[3] V. Boekelheide , G. K. Vick , J. Am. Chem. Soc. 1956, 78, 653–658.

[4a] C. Chaolumen , I. A. Stepek , K. E. Yamada , H. Ito , K. Itami , Angew. Chem. Int. Ed. 2021, 60, 23508–23532; 10.1002/anie.202100260 33547701

[4b] S. H. Pun , Q. Miao , Acc. Chem. Res. 2018, 51, 1630–1642; 29974752 10.1021/acs.accounts.8b00140

[4c] M. A. Majewski , M. Stępień , Angew. Chem. Int. Ed. 2019, 58, 86–116; 10.1002/anie.201807004 30006951

[4d] K. M. Magiera , V. Aryal , W. A. Chalifoux , Org. Biomol. Chem. 2020, 18, 2372–2386. 32196052 10.1039/d0ob00182a

[5] K. Yamamoto , T. Harada , Y. Okamoto , H. Chikamatsu , M. Nakazaki , Y. Kai , T. Nakao , M. Tanaka , S. Harada , N. Kasai , J. Am. Chem. Soc. 1988, 110, 3578–3584.

[6a] K. Kawasumi , Q. Zhang , Y. Segawa , L. T. Scott , K. Itami , Nat. Chem. 2013, 5, 739–744; 23965674 10.1038/nchem.1704

[6b] A. Pradhan , P. Dechambenoit , H. Bock , F. Durola , J. Org. Chem. 2013, 78, 2266–2274; 23374076 10.1021/jo3027752

[6c] K. Kato , Y. Segawa , L. T. Scott , K. Itami , Chem. Asian J. 2015, 10, 1635–1639; 26062779 10.1002/asia.201500560

[6d] J. M. Fernandez-Garcia , P. J. Evans , S. Medina Rivero , I. Fernandez , D. Garcia-Fresnadillo , J. Perles , J. Casado , N. Martin , J. Am. Chem. Soc. 2018, 140, 17188–17196; 30431273 10.1021/jacs.8b09992

[6e] K. Kato , K. Takaba , S. Maki-Yonekura , N. Mitoma , Y. Nakanishi , T. Nishihara , T. Hatakeyama , T. Kawada , Y. Hijikata , J. Pirillo , L. T. Scott , K. Yonekura , Y. Segawa , K. Itami , J. Am. Chem. Soc. 2021, 143, 5465–5469; 33759524 10.1021/jacs.1c00863

[6f] X. Yang , F. Rominger , M. Mastalerz , Angew. Chem. Int. Ed. 2019, 58, 17577–17582; 10.1002/anie.201908643 PMC6899884 31550407

[6g] J. Ma , Y. Fu , E. Dmitrieva , F. Liu , H. Komber , F. Hennersdorf , A. A. Popov , J. J. Weigand , J. Liu , X. Feng , Angew. Chem. Int. Ed. 2020, 59, 5637–5642; 10.1002/anie.201914716 PMC7155134 31867754

[6h] T. Fujikawa , Y. Segawa , K. Itami , J. Org. Chem. 2017, 82, 7745–7749. 28686025 10.1021/acs.joc.7b01540

[7a] A. Konishi , K. Horii , D. Shiomi , K. Sato , T. Takui , M. Yasuda , J. Am. Chem. Soc. 2019, 141, 10165–10170; 31132260 10.1021/jacs.9b04080

[7b] Y. Fei , Y. Fu , X. Bai , L. Du , Z. Li , H. Komber , K.-H. Low , S. Zhou , D. L. Phillips , X. Feng , J. Liu , J. Am. Chem. Soc. 2021, 143, 2353–2360. 33502182 10.1021/jacs.0c12116

[8a] K. Y. Cheung , X. Xu , Q. Miao , J. Am. Chem. Soc. 2015, 137, 3910–3914; 25741577 10.1021/jacs.5b00403

[8b] X. Gu , H. Li , B. Shan , Z. Liu , Q. Miao , Org. Lett. 2017, 19, 2246–2249; 28421768 10.1021/acs.orglett.7b00714

[8c] K. Y. Cheung , Q. Miao , Chin. Chem. Lett. 2019, 30, 1506–1508.

[9] S. H. Pun , C. K. Chan , J. Luo , Z. Liu , Q. Miao , Angew. Chem. Int. Ed. 2018, 57, 1581–1586; 10.1002/anie.201711437 29251395

[10] V. Akhmetov , A. Förtsch , M. Feofanov , S. Troyanov , K. Amsharov , Org. Chem. Front. 2020, 7, 1271–1275.

[11] K. Kawai , K. Kato , L. Peng , Y. Segawa , L. T. Scott , K. Itami , Org. Lett. 2018, 20, 1932–1935. 29560728 10.1021/acs.orglett.8b00477

[12] W. C. Fu , Z. Wang , W. T. K. Chan , Z. Lin , F. Y. Kwong , Angew. Chem. Int. Ed. 2017, 56, 7166–7170; 10.1002/anie.201703551 28510348

[13a] K. Kantarod , T. Worakul , D. Soorukram , C. Kuhakarn , V. Reutrakul , P. Surawatanawong , W. Wattanathana , P. Leowanawat , Org. Chem. Front. 2021, 8, 522–530;

[13b] X. Li , J.-W. Han , Y.-X. Zhang , H. N. C. Wong , Asian J. Org. Chem. 2017, 6, 1876–1884.

[14] J. Yan , M. S. Rahman , N. Yoshikai , Chem. Eur. J. 2018, 25, 9395–9399. 30457177 10.1002/chem.201805746

[15] I. R. Marquez , N. Fuentes , C. M. Cruz , V. Puente-Munoz , L. Sotorrios , M. L. Marcos , D. Choquesillo-Lazarte , B. Biel , L. Crovetto , E. Gomez-Bengoa , M. T. Gonzalez , R. Martin , J. M. Cuerva , A. G. Campana , Chem. Sci. 2017, 8, 1068–1074. 28451246 10.1039/c6sc02895k PMC5357993

[16] J. M. Farrell , V. Grande , D. Schmidt , F. Würthner , Angew. Chem. Int. Ed. 2019, 58, 16504–16507; 10.1002/anie.201909975 PMC6900032 31507020

[17] C. Zhu , K. Shoyama , F. Würthner , Angew. Chem. Int. Ed. 2020, 59, 21505–21509; 10.1002/anie.202010077 PMC7756343 32815658

[18] M. Schnitzlein , C. Mützel , K. Shoyama , J. M. Farrell , F. Würthner , Eur. J. Org. Chem. 2022, 2022, e202101273.

[19a] J. M. Farrell , D. Schmidt , V. Grande , F. Würthner , Angew. Chem. Int. Ed. 2017, 56, 11846–11850; 10.1002/anie.201706346 28741895

[19b] J. M. Farrell , C. Mützel , D. Bialas , M. Rudolf , K. Menekse , A. M. Krause , M. Stolte , F. Würthner , J. Am. Chem. Soc. 2019, 141, 9096–9104. 31117551 10.1021/jacs.9b04675

[20] Deposition Number(s) 2183072 (for **1**), 2183070 (**3**) and 2183071 (**4**) contain(s) the supplementary crystallographic data for this paper. These data are provided free of charge by the joint Cambridge Crystallographic Data Centre and Fachinformationszentrum Karlsruhe Access Structures service.

[21] F. H. Allen , O. Kennard , D. G. Watson , L. Brammer , A. G. Orpen , R. Taylor , J. Chem. Soc. Perkin Trans. 2 1987, S1–S19.

[22] Z. Chen , C. S. Wannere , C. Corminboeuf , R. Puchta , P. v. R. Schleyer , Chem. Rev. 2005, 105, 3842–3888. 16218569 10.1021/cr030088+

[23] D. Geuenich , K. Hess , F. Köhler , R. Herges , Chem. Rev. 2005, 105, 3758–3772. 16218566 10.1021/cr0300901

[24] M. Antić , B. Furtula , S. Radenković , J. Phys. Chem. A 2017, 121, 3616–3626. 28440657 10.1021/acs.jpca.7b02521

