# Supplementary material for: π‐Extended Pleiadienes by [5+2] Annulation of 1‐Boraphenalenes and ortho‐Dihaloarenes
Source: Chemistry. 2022 Aug 31;28(61):e202202053. doi: 10.1002/chem.202202053 (PMC9804371; doi:10.1002/chem.202202053)
Supplement: Supplementary file 1 — Supporting Information [file CHEM-28-0-s001.pdf]

Chemistry–A European Journal

Supporting Information

π-Extended Pleiadienes by [5+2] Annulation of 1-Boraphenalenes and *ortho*-Dihaloarenes

Matthias Schnitzlein, Chongwei Zhu, Kazutaka Shoyama, and Frank Würthner*

Table of Contents

1. Materials and methods 2
2. Experimental procedures 3
3. NMR Spectra 5
4. Cyclic voltammetry 11
5. UV/Vis absorption and fluorescence spectra 12
6. Crystal structure data 14
7. Computational data 16
7.1. NICS data 16
7.2 Optimized structures 17
8. References 23

1. Materials and methods

General remarks: Commercially available chemicals were purchased and directly used without further purification. Preparative column chromatography was performed with glass columns of various sizes packed with silica-gel from Macherey-Nagel (particle size 40–63 µm) as stationary phase. Preparative thin-layer chromatography was carried out with ALUGRAM Xtra SIL G plates from Macherey-Nagel. Dichloromethane was distilled before use as mobile phase. 1,3-Diisopropylimidazol-2-ylidene borane[1], 2-phenyl-1*H*-naphtho[1,8-*bc*]borinin-1-ol (**9a**)[2], (*E*)-1,2-di(naphthalen-1-yl)ethene[3] and (*E*)-1-styrylpyrene[4] were prepared using modified literature-reported methods. All other reagents and solvents were obtained from commercial sources and used without further purification. Dry dichloromethane was obtained from a solvent purification system PS-M6-6/7 from inert technologies. All [5+2] annulation reactions were carried out in dry Schlenk tubes under nitrogen atmosphere.

UV/Vis absorption spectroscopy was carried out on a Jasco V-670 spectrophotometer with 1 cm Hellma quartz glass cuvettes and spectroscopy grade solvent.

Fluorescence spectra were recorded on a FLS980 spectrometer from Edinburgh Instruments with 1 cm Hellma quartz glass cuvettes. Quantum yields were measured using a Hamamatsu Ulbricht sphere A9924-01 with a continuous Xe lamp and a Hamamatsu photonic multi-channel analyser C10027 and are uncorrected.

NMR spectroscopy was measured on Bruker Avance III HD 400 spectrometers. Chemical shifts are given in ppm relative to tetramethylsilane and calibrated with regard to the residual solvent signal of DMSO-d_6 (δ(^{1}H) = 2.50 ppm, δ(^{13}C) = 39.52 ppm) and CD_2Cl_2 (δ(^{1}H) = 5.32 ppm, δ(^{13}C) = 53.84 ppm).[5] The multiplicities are given as abbreviations (s = singlet, d = doublet, t = triplet, m = multiplet), coupling constants (*J*) are shown in Hertz (Hz).

Mass spectra were measured on a Bruker Daltonics ultrafleXtreme mass spectrometer (matrix-assisted laser desorption/ionisation time-of-flight, MALDI-TOF) using *trans*-2-[3-(4-tert-butylphenyl)-2-methyl-2-propenylidene]malononitrile (DCTB) as matrix.

Cyclic and differential pulse voltammetry were measured with a standard commercial electrochemical analyser (EC epsilon; BAS Instruments, UK) with a three-electrode single-compartment cell. Tetrabutylammonium hexafluorophosphate ((n-Bu)$_4$NPF$_6$) was applied as supporting electrolyte with ferrocene (Fc) as an internal standard for the calibration of potentials, Ag/AgCl as reference electrode, Pt disc and Pt wire as working and auxiliary electrodes, respectively. Cyclic voltammetry (CV) and differential pulse voltammetry (DPV) were performed at a scan rate of 100 mV/s at room temperature under argon atmosphere.

Single crystal X-ray analysis was carried out on Bruker D8 Quest Kappa diffractometers with a PhotonII CMOS detector and multi-layered mirror monochromated CuKα radiation.

Melting points were measured with a polarisation microscope BX41 from Olympus in conjunction with a temperature control element TP-94 by Linkam. All given melting points are uncorrected.

Theoretical calculations: Geometry optimizations, NICS(1)$_{zz}$ values, excited state, and AICD calculations were performed using density functional theory (DFT) at the level of B3LYP/6-31+G(d) as implemented in the Gaussian 09 program.[6] The AICD software package for visualisation was kindly provided by Prof. R. Herges. For preparation and analysis of NICS(1)$_{zz}$ values for the non-planar systems as well as for plotting the frontier orbitals the multiwfn package was employed.[7]

2. Experimental procedures

2-(Naphthalen-1-yl)-1*H*-naphtho[1,8-*bc*]borinin-1-ol

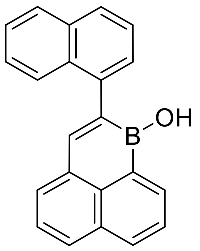

9b

In a nitrogen-filled glovebox 1,3-diisopropylimidazol-2-ylidene borane (200 mg, 1.20 mmol, 1.2 equiv.) was weighed into a 50 mL round bottom flask with a magnetic stirring bar and dissolved in 5 mL dry chlorobenzene. Then $HNTf_2$ (344 mg, 1.22 mmol, 1.22 equiv.) and additional dry chlorobenzene (2 mL) was added, whereupon hydrogen evolved. The reaction mixture was then stirred for 90 min. Afterwards (*E*)-1,2-di(naphthalen-1-yl)ethene (281 mg, 1.00 mmol, 1.0 equiv.) and dry chlorobenzene (5 mL) was added, and a 50 mL dropping funnel was fixed onto the flask. In the dropping funnel a solution of TEMPO (361 mg, 2.30 mmol, 2.3 equiv.) in dry chlorobenzene (5 mL) was prepared and the apparatus sealed with a glass stopper. The apparatus was then removed from the glovebox and stirred at 110 °C for 5 h. Then the solution was cooled to 80 °C and the TEMPO solution in the dropping funnel was added. The mixture was stirred at 80 °C for 36 h. After cooling down to room temperature the volatiles were evaporated under reduced pressure and the residue was purified by silica-gel column chromatography (eluent 1:1 *n*-hexane:dichloromethane by volume).

Yield: 132 mg (432 µmol, 43%) yellow solid. **^{1}H NMR** (400 MHz, DMSO-d_6, 298 K): δ (ppm) = 9.52 (s, 1H), 8.67 (dd, J = 7.0, 1.1 Hz, 1H), 8.29 (dd, J = 8.2, 1.1 Hz, 1H), 8.08 (dd, J = 8.3, 0.9 Hz), 7.95 (d, J = 7.9 Hz, 1H), 7.90–7.85 (m, 2H), 7.84–7.79 (m, 3H), 7.63 (t, J = 7.7 Hz, 1H), 7.55 (t, J = 7.7 Hz, 1H), 7.50 (t, J = 7.5 Hz, 1H), 7.44–7.39 (m, 2H). **^{13}C{^{1}H} NMR** (101 MHz, CD_2Cl_2, 298 K): δ (ppm) = 148.2, 144.0, 141.8, 135.4, 133.3, 133.2, 132.1, 131.8, 131.6, 131.4, 131.3, 131.1, 130.6, 128.0, 126.5, 126.4, 126.2, 126.0, 125.7, 125.5, 125.3, 125.1. **HRMS** (MALDI-TOF/DCTB): *m/z* $[M]^+$ calculated for $C_{22}H_{15}BO$: 306.1216, *m/z* found: 306.1212. **Melting point:** 65–67 °C.

4-Phenyl-5*H*-pyreno[10,1-*bc*]borinin-5-ol

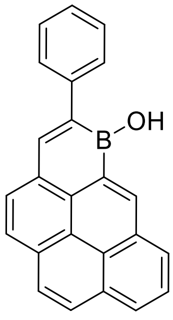

10

In a nitrogen-filled glovebox 1,3-diisopropylimidazol-2-ylidene borane (400 mg, 2.40 mmol, 1.2 equiv.) was weighed into a 100 mL round bottom flask with a magnetic stirring bar and dissolved in 10 mL dry chlorobenzene. Then $HNTf_2$ (686 mg, 2.44 mmol, 1.22 equiv.) and additional dry chlorobenzene (4 mL) was added, whereupon hydrogen evolved. The reaction mixture was then stirred for 90 min. Afterwards (*E*)-1-styrylpyrene (608 mg, 2.00 mmol, 1.0 equiv.) and dry chlorobenzene (10 mL) was added, and a 50 mL dropping funnel was fixed onto the flask. In the dropping funnel a solution of TEMPO (722 mg, 4.60 mmol, 2.3 equiv.) in dry chlorobenzene (10 mL) was prepared and the apparatus sealed with a glass stopper. The apparatus was then removed from the glovebox and stirred at 110 °C for 5 h. Then the solution was cooled to 80 °C and the TEMPO solution in the dropping funnel added. The mixture was stirred at 80 °C for 36 h. After cooling down to room temperature the volatiles were evaporated under reduced pressure and the residue was purified by silica-gel column chromatography (gradient elution from 2:1 toluene:dichloromethane to 3:2 by volume).

Yield: 276 mg (0.520 mmol, 42%) orange solid. **^{1}H NMR** (400 MHz, DMSO-d_6, 298 K): δ (ppm) = 10.13 (s, 1H), 9.50 (s, 1H), 8.53 (t, J = 8.1 Hz, 2H), 8.39–8.28 (m, 2H), 8.26 (s, 2H), 8.24–8.17 (m, 2H), 7.79 (d, J = 6.8 Hz, 2H), 7.46 (t, J = 7.6 Hz, 2H), 7.34 (d, J = 7.3 Hz, 1H). **^{13}C{^{1}H} NMR** (101 MHz, DMSO-d_6, 298 K): δ (ppm) = 147.5, 142.5, 138.9, 131.5, 130.5, 129.8, 129.7, 129.5, 128.9, 128.6, 128.5, 128.23, 128.16, 127.9, 127.8, 127.7, 127.3, 126.8, 126.5, 125.3, 124.8, 124.2, 122.9. **HRMS** (MALDI-TOF/DCTB): *m/z* $[M+H]^+$ calculated for $C_{24}H_{15}BO$: 531.1294, found: 531.1289. **Melting point:** 129–130 °C.

8-(Naphthalen-1-yl)cyclohepta[1,2-*b*:3,4,5-*d'e'*]dinaphthalene

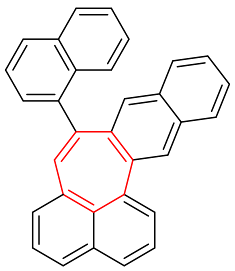

1

In a Schlenk tube equipped with a magnetic stirring bar were weighed in **9b** (31 mg, 0.10 mmol, 1.0 equiv.), 2,3-dibromonaphthalene (57 mg, 0.20 mmol, 2.0 equiv.), $[Pd_2(dba)_3]\cdot CHCl_3$ (1.6 mg, 1.5 µmol, 1.5 mol%), $P(^tBu)_3\cdot HBF_4$ (1.0 mg, 3.6 µmol, 3.6 mol%) and cesium carbonate (0.11 g, 0.33 mmol, 3.3 equiv.). Under nitrogen atmosphere distilled water (18 µL, 1.0 mmol, 10 equiv.) and *tert*-amyl alcohol (3 mL) were added by syringe. The mixture was stirred at room temperature for 1 h and then at 100 °C for 42 h. It was cooled to room temperature, filtered through celite and the filter cake was washed with ethyl acetate. After evaporation of the solvent from the filtrate the residue was purified by silica-gel column and preparative thin-layer chromatography (eluent *n*-hexane:ethyl acetate 19:1 by volume) followed by washing with methanol.

Yield: 18.2 mg (45.0 µmol, 45%) yellow solid. **^{1}H NMR** (400 MHz, CD_2Cl_2, 298 K): δ (ppm) = 8.07–8.05 (dd, J = 8.5, 1.0 Hz, 1H), 7.97–7.92 (m, 2H), 7.90 (dd, J = 7.3, 1.4 Hz, 1H), 7.84 (dd, J = 8.1, 1.1 Hz, 1H), 7.80–7.78 (m, 1H), 7.74 (s, 1H), 7.73–7.69 (m, 2H), 7.67–7.62 (m, 2H), 7.47–7.27 (m, 7H), 7.17 (s, 1H), 6.86 (s, 1H). **^{13}C{^{1}H} NMR** (101 MHz, CD_2Cl_2, 298 K): δ (ppm) = 141.9, 139.9, 139.1, 138.8, 137.5, 137.3, 136.4, 134.7, 134.4, 134.3, 134.01, 133.99, 132.4, 132.0, 130.7, 130.2, 128.6, 128.3, 127.9, 127.67, 127.66, 127.6, 127.3, 126.6, 126.52, 126.47, 126.3, 126.1, 125.9, 125.8, 125.7. **HRMS** (MALDI-TOF/DCTB): *m/z*: $[M]^+$ calculated for $C_{32}H_{20}$: 404.1560, found: 404.1560. **UV/Vis** (CH_2Cl_2, 298 K): λ = 300 nm (ε = 17400 L mol^{-1} cm^{-1}), 378 nm (ε = 8960 L mol^{-1} cm^{-1}). **Fluorescence**

(CH_2Cl_2, 298 K): λ_{em} = 474 nm, Φ_{em} = 0.23. **Cyclic voltammetry** ($c \approx 2.5\times10^{-4}$ M, 0.1 M n-Bu_4NPF_6 in CH_2Cl_2 at 298 K, E_{Redox} vs. Fc^+/Fc): E_{ox1} = 0.72 V. **Melting point:** 110–112 °C.

Dibenzo[*c*,*g*]naphtho[1',8':5,6,7]cyclohepta[1,2,3-*jk*]fluorene

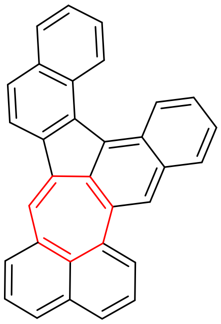

2

In a Schlenk tube equipped with a magnetic stirring bar were weighed in **1** (12 mg, 30 µmol, 1.0 equiv.) and DDQ (6.7 mg, 30 µmol, 1.0 equiv.). Under nitrogen atmosphere the solids were dissolved in dry dichloromethane (15 mL). After cooling to 0 °C triflic acid (0.15 mL) was added, whereupon the reaction mixture turned black. After stirring for 15 min at 0 °C the reaction was quenched by addition of saturated aqueous sodium bicarbonate solution. The phases were separated, the aqueous phase extracted with dichloromethane (2 × 20 mL) and the combined organic phases washed with water (3 × 25 mL) before drying over magnesium sulfate. The crude was then purified by silica-gel column chromatography (eluent cyclohexane:dichloromethane 4:1 by volume) and washed with methanol.

Yield: 4.6 mg (11.9 µmol, 39%) red solid. **^{1}H NMR** (400 MHz, CD_2Cl_2, 298 K) δ (ppm) = 8.74 (d, J = 8.6 Hz, 1H), 8.55 (d, J = 8.6 Hz, 1H), 8.51 (d, J = 8.7 Hz, 1H), 8.09 (s, 1H), 7.91–7.99 (m, 3H), 7.76–7.84 (m, 3H), 7.68 (s, 1H), 7.58–7.63 (m, 4H), 7.42–7.53 (m, 3H). **^{13}C{^{1}H} NMR** (101 MHz, CD_2Cl_2, 298 K) δ (ppm) = 140.0, 138.91, 138.87, 137.9, 137.6, 137.2, 136.3, 135.7, 135.2, 134.9, 134.6, 133.9, 133.8, 133.3, 131.5, 131.4, 131.0, 130.0, 139.8, 129.7, 129.4, 129.3, 128.8, 127.62, 127.57, 127.5, 126.1, 126.0, 125.4, 124.7, 124.2, 121.7. **HRMS** (MALDI-TOF/DCTB): m/z: $[M]^+$ calculated for $C_{32}H_{18}$: 402.1404, found: 402.1405 **UV/Vis** (CH_2Cl_2, 298 K): λ = 441 nm (ε = 14100 L mol^{-1} cm^{-1}), 320 nm (ε = 24900 L mol^{-1} cm^{-1}), 277 nm (ε = 49300 L mol^{-1} cm^{-1}). **Cyclic voltammetry** ($c \approx 2.5\times10^{-4}$ M, 0.1 M n-Bu_4NPF_6 in CH_2Cl_2 at 298 K, E_{Redox} vs. Fc^+/Fc): E_{ox1} = 0.55 V. **Melting point:** 232–234 °C.

8,17-Diphenylbenzo[1,2-*i*:4,5-*i'*]dipleiadiene

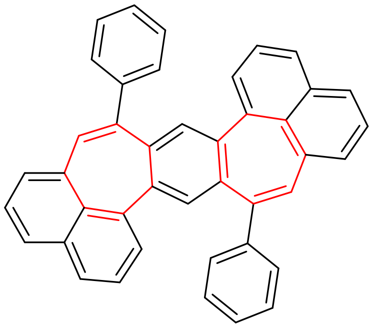

3

In a Schlenk tube equipped with a magnetic stirring bar were weighed in **9a** (65.0 mg, 254 µmol, 2.4 equiv.), 1,4-dibromo-2,5-diiodobenzene (51.6 mg, 106 µmol, 1.0 equiv.), $[Pd_2(dba)_3]\cdot CHCl_3$ (6.3 mg, 6.3 µmol, 6 mol%), $P(^tBu)_3\cdot HBF_4$ (4.3 mg, 14.8 µmol, 14 mol%) and cesium carbonate (227 mg, 698 µmol, 6.6 equiv.). Under nitrogen atmosphere distilled water (38.1 µL, 2.11 mmol, 20 equiv.) and *tert*-amyl alcohol (12 mL) were added by syringe. The mixture was stirred at room temperature for one hour and then at 100 °C for 42 h. It was cooled to room temperature, filtered through Celite and the filter cake was washed with ethyl acetate and dichloromethane. After evaporation of the solvent from the filtrate the residue was purified by two-time column chromatography on silica-gel (first eluent *n*-hexane:ethyl acetate 10:1 by volume, second eluent *n*-hexane:dichloromethane 4:1 by volume).

Yield: 11.2 mg (21.2 µmol, 20%) yellow solid. **^{1}H NMR** (400 MHz, CD_2Cl_2, 298 K): δ (ppm) = 7.61 (dd, J = 8.2, 1.3 Hz, 2H), 7.57 (dd, J = 8.1, 1.4 Hz, 2H), 7.54–7.51 (m, 4H), 7.47 – 7.42 (m, 4H), 7.40–7.35 (m, 2H), 7.35–7.25 (m, 6H), 7.21 (dd, J = 7.4, 1.3 Hz, 2H), 6.78 (s, 2H), 6.70 (s, 2H). **^{13}C{^{1}H} NMR** (101 MHz, CD_2Cl_2, 298 K) δ (ppm) = 144.0, 141.3, 140.4, 139.9, 138.3, 137.4, 136.8, 136.6, 134.5, 130.0, 129.9,128.8, 128.5, 128.2, 128.0, 127.3, 127.1, 126.3, 125.7. **HRMS** (MALDI-TOF/DCTB): m/z ($[M^+]$) calculated for $C_{42}H_{26}$: 530.2030, found: 530.2225. **UV/Vis** (CH_2Cl_2, 298 K): λ = 402 nm (ε = 20600 L mol^{-1} cm^{-1}), 328 nm (ε = 33000 L mol^{-1} cm^{-1}), 298 nm (ε = 32400 L mol^{-1} cm^{-1}). **Fluorescence** (CH_2Cl_2, 298 K): λ_{em} = 558 nm, Φ_{em} = 0.07. **Cyclic voltammetry** ($c \approx 2.5\times10^{-4}$ M, 0.1 M n-Bu_4NPF_6 in CH_2Cl_2 at 298 K, E_{redox} *vs.* Fc^+/Fc): E_{ox1} = 0.57 V, E_{ox2}= 0.89 V. **Melting point:** .320–321 °C.

9,20-Diphenylbenzo[1'',2'':9,10;4'',5'':9',10']bis(pleiadieno[2,3,3a,4-*ija*]naphthalene)

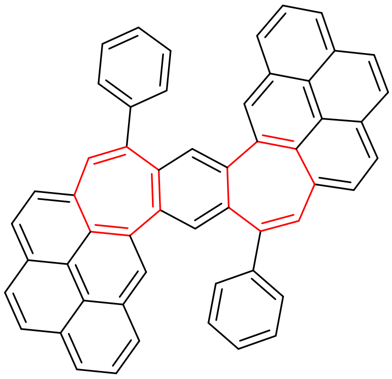

4

In a Schlenk tube equipped with a magnetic stirring bar were weighed in **10** (40 mg, 0.12 mmol, 2.4 equiv.), 1,4-dibromo-2,5-diiodobenzene (25 mg, 50 µmol, 1.0 equiv.), $[Pd_2(dba)_3]\cdot CHCl_3$ (3.1 mg, 3.0 µmol, 6.0 mol%), $P(^tBu)_3\cdot HBF_4$ (2.1 mg, 7.1 µmol, 14 mol%) and cesium carbonate (109 mg, 333 µmol, 6.6 equiv.). Under nitrogen atmosphere distilled water (19 µL, 1.0 mmol, 20 equiv.) and *tert*-amyl alcohol (12 mL) were added by syringe. The mixture was stirred at room temperature for 1 h and then at 100 °C for 42 h. It was cooled to room temperature, filtered through celite and the filter cake was washed with ethyl acetate and dichloromethane. After evaporation of the solvent from the filtrate the residue was purified by silica-gel column chromatography (first eluent *n*-hexane:ethyl acetate 10:1 by volume, second eluent *n*-hexane:dichloromethane 4:1 by volume).

Yield: 3.4 mg (5.1 µmol, 10%) red solid. **^{1}H NMR** (400 MHz, CD_2Cl_2, 298 K) δ (ppm) = 8.08–8.03 (m, 4H), 7.97 (d, J = 2.7 Hz, 4H), 7.94–7.91 (m, 4H), 7.83 (s, 2H), 7.81 (d, J = 8.6 Hz, 2H), 7.68 (dt, J = 8.1, 1.8 Hz, 4H), 7.51 (t, J = 7.6 Hz, 4H), 7.45–7.38 (m, 2H), 7.07 (s, 2H), 6.97 (s, 2H). **^{13}C NMR** signals could not be observed due to low solubility. **HRMS** (MALDI-TOF/DCTB): m/z: $[M]^+$ calculated for $C_{54}H_{30}$: 678.2343, found: 678.2345. **UV/Vis** (CH_2Cl_2, 298 K): λ = 437 nm (ε = 20500 L mol^{-1} cm^{-1}), 345 nm (ε = 26100 L mol^{-1} cm^{-1}), 302 nm (ε = 49900 L mol^{-1} cm^{-1}). **Fluorescence** (CH_2Cl_2, 298 K): λ_{em} = 624 nm, Φ_{em} = 0.02. **Cyclic voltammetry** ($c \approx 2.5\times10^{-4}$ M, 0.1 M n-Bu_4NPF_6 in CH_2Cl_2 at 298 K, E_{Redox} vs. Fc^+/Fc): E_{ox1} = 0.47 V, E_{ox2} = 0.69 V. **Melting point:** >350 °C.

3. NMR Spectra

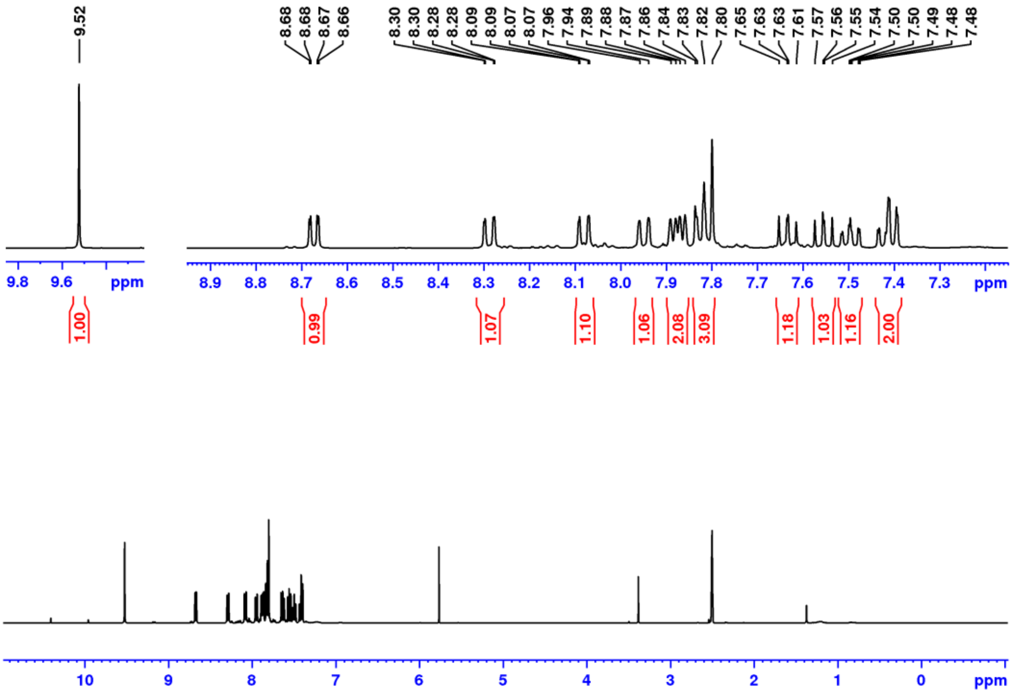

Figure S1. ^{1}H NMR (400 MHz, 298 K) spectrum of **9b** in DMSO-d_6.

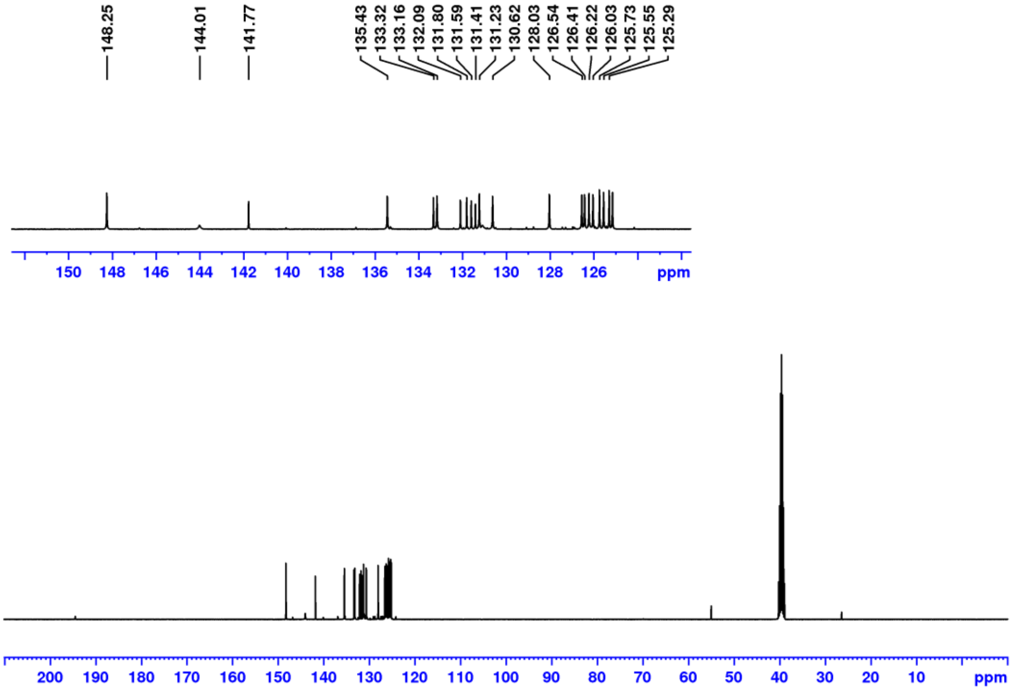

Figure S2. ^{13}C NMR (101 MHz, 298 K) spectrum of **9b** in DMSO-d_6.

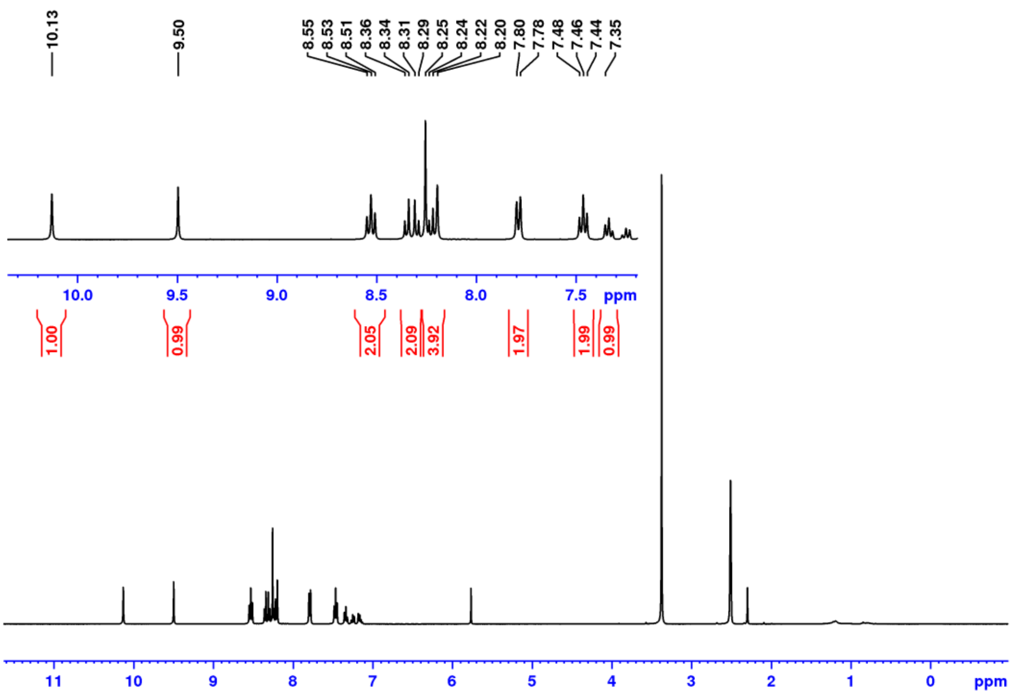

Figure S3. ^{1}H NMR (400 MHz, 298 K) spectrum of **10** in DMSO-d_6.

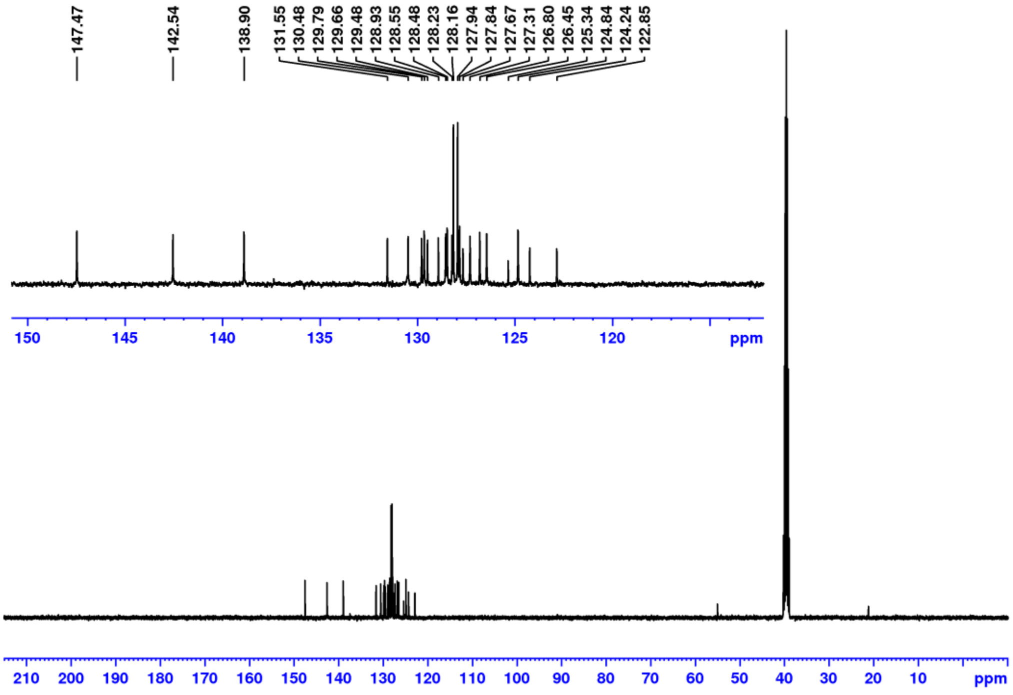

Figure S4. ^{13}C NMR (101 MHz, 298 K) spectrum of **10** in DMSO-d_6.

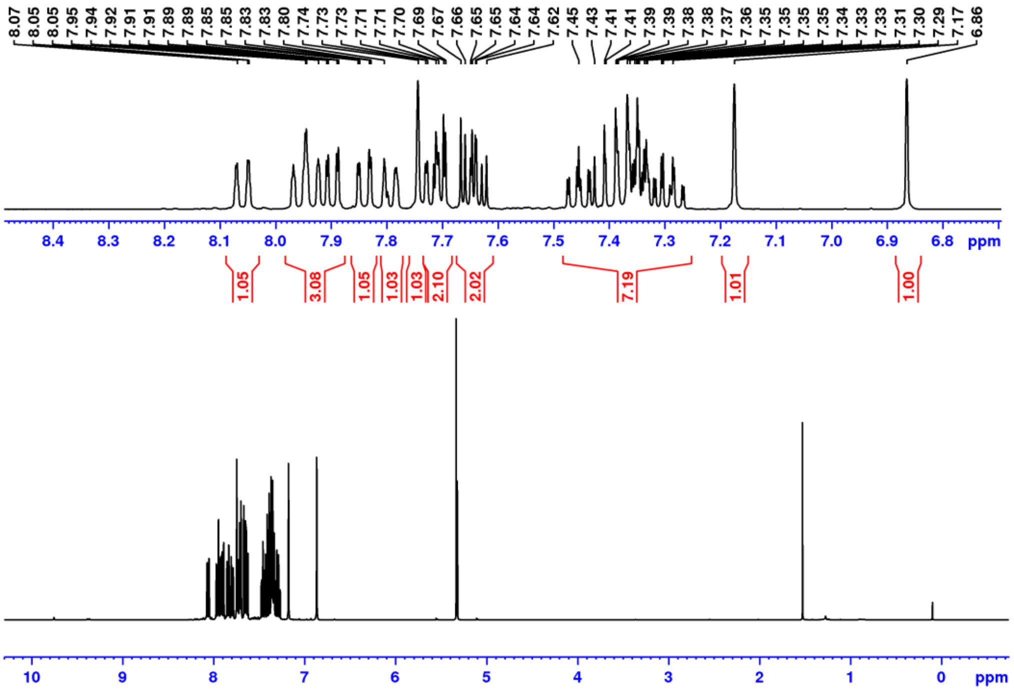

Figure S5. ^{1}H NMR (400 MHz, 298 K) spectrum of **1** in CD_2Cl_2.

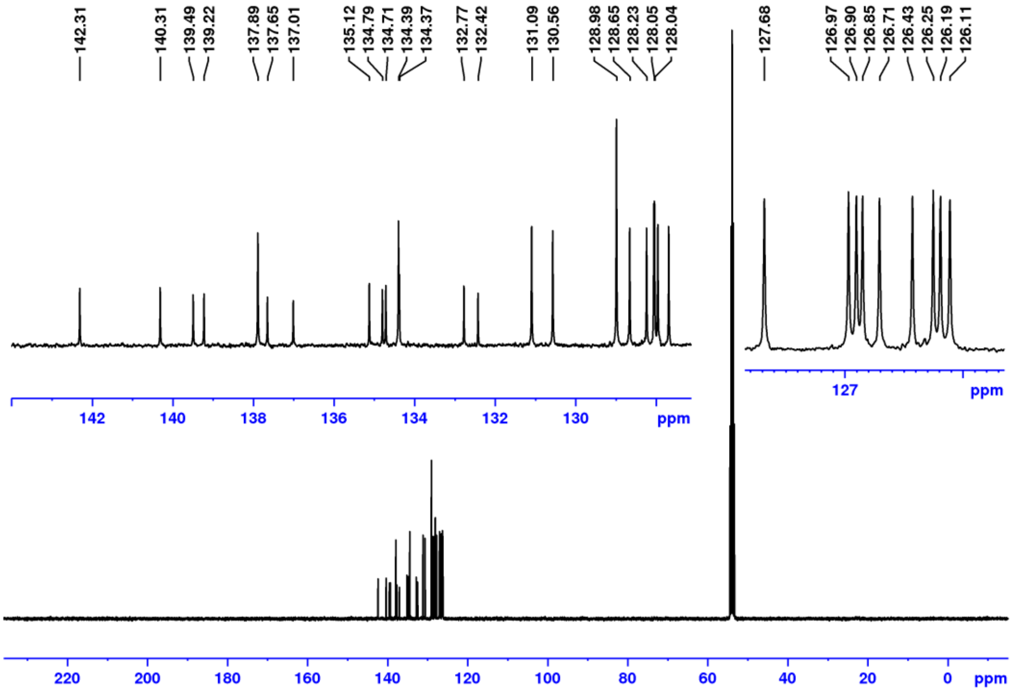

Figure S6. ^{13}C NMR (101 MHz, 298 K) spectrum of **1** in CD_2Cl_2.

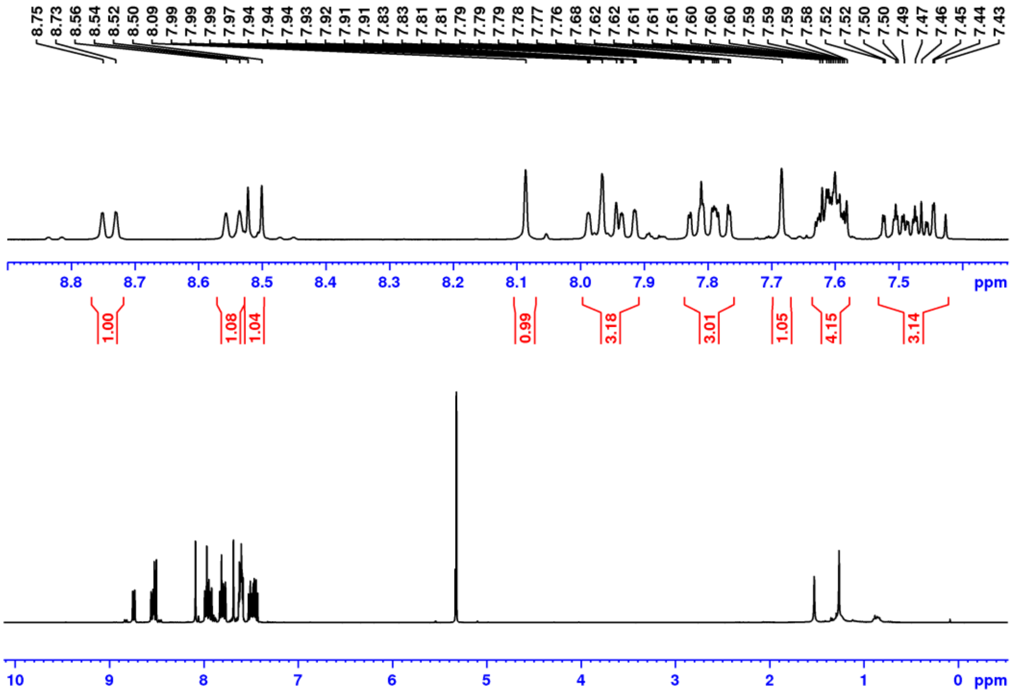

Figure S7. ^{1}H NMR (400 MHz, 298 K) spectrum of **2** in CD_2Cl_2.

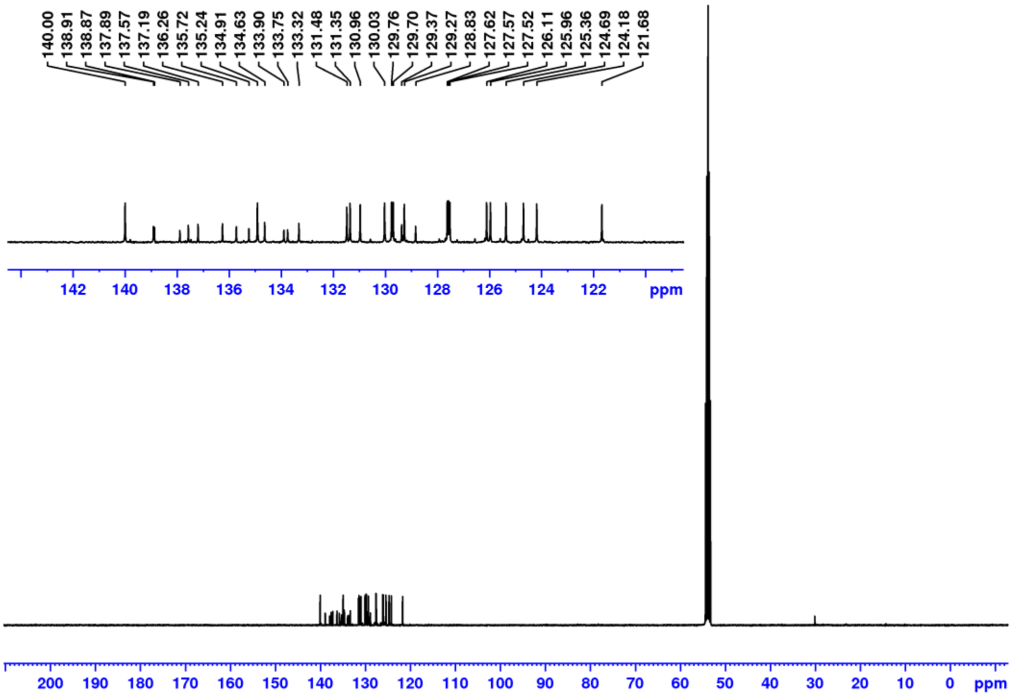

Figure S8. ^{13}C NMR (101 MHz, 298 K) spectrum of **2** in CD_2Cl_2.

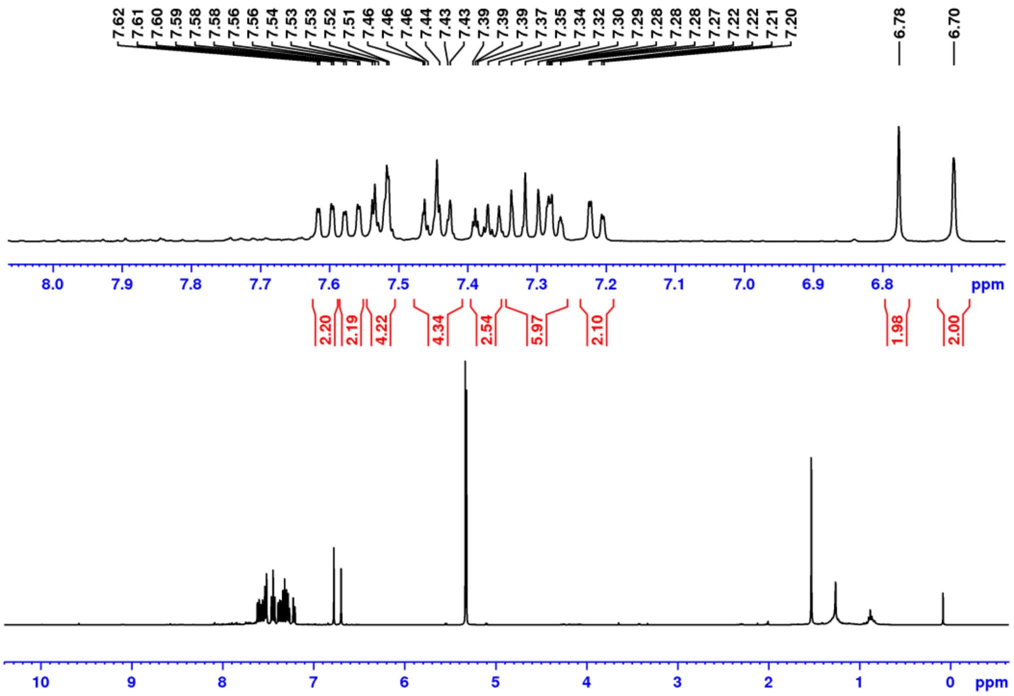

Figure S9. ^{1}H NMR (400 MHz, 298 K) spectrum of **3** in CD_2Cl_2.

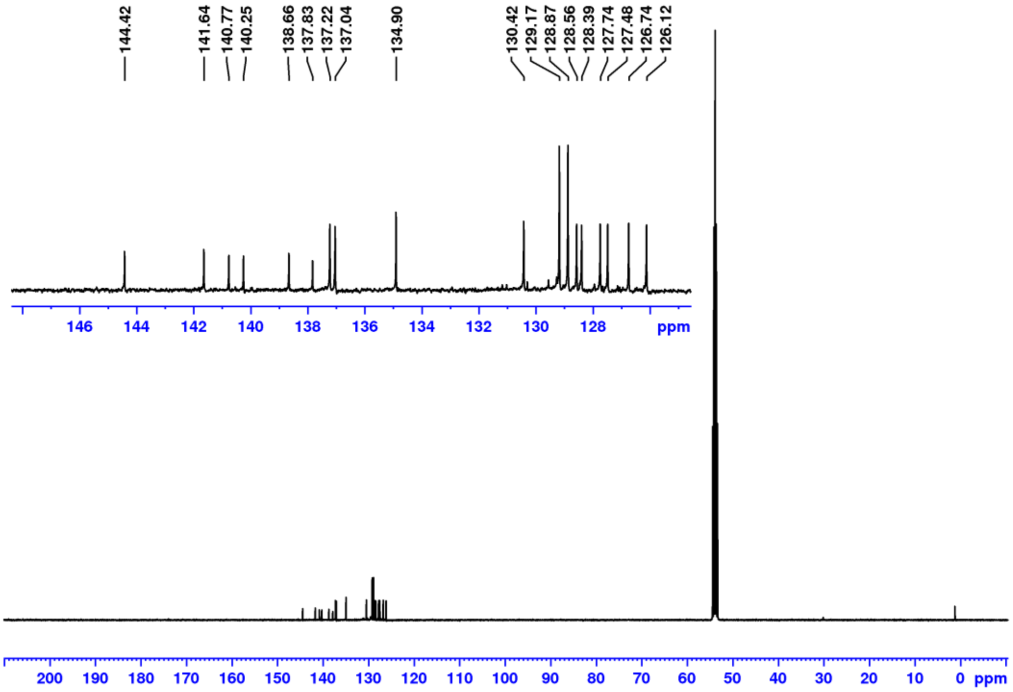

Figure S10. ^{13}C NMR (101 MHz, 298 K) spectrum of **3** in CD_2Cl_2.

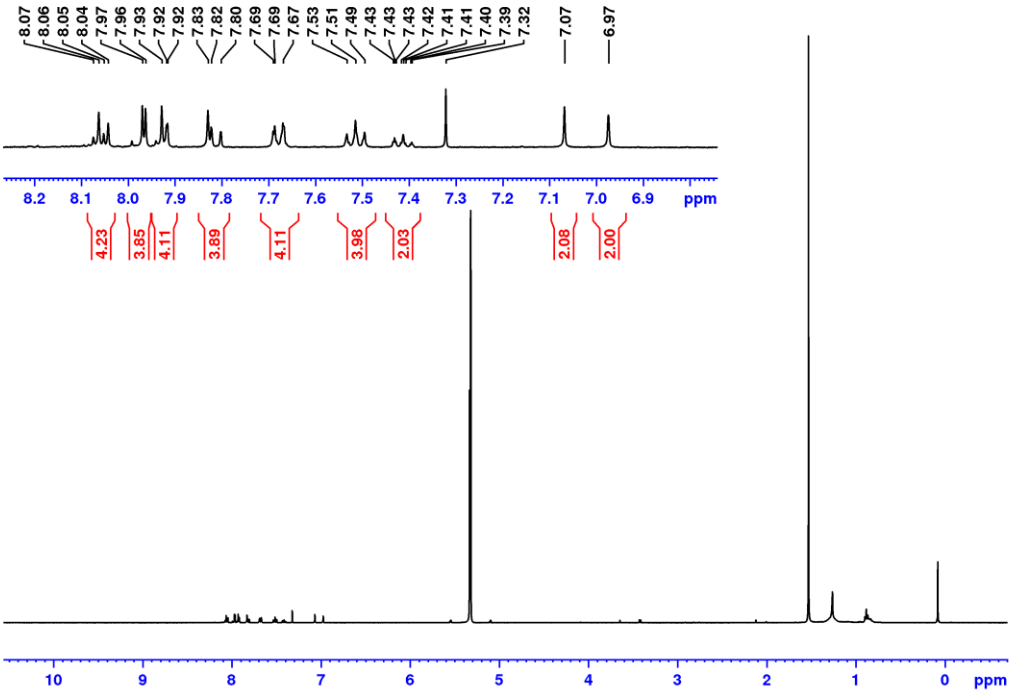

Figure S11. ^{1}H NMR (400 MHz, 298 K) spectrum of **4** in CD_2Cl_2.

4. Cyclic voltammetry

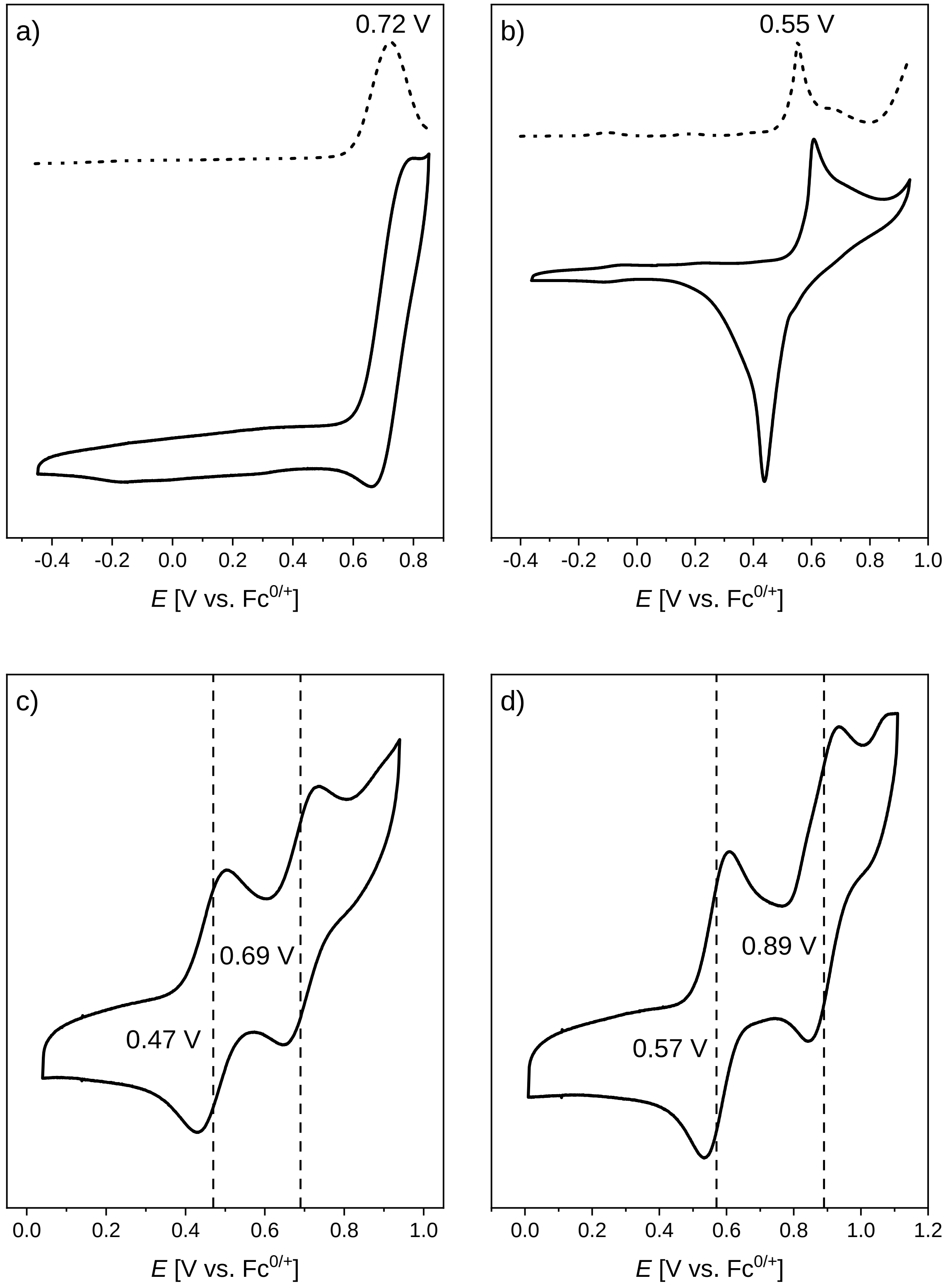

Figure S12. Cyclic voltammograms (solid lines) of **1–4** (a-d) taken at 298 K in dry, degassed dichloromethane ($c \approx 2.5\times10^{-4}$ M) with 0.1 M (n-Bu)$_4$NPF$_6$ under argon atmosphere at a scan rate of 100 mV/s. For a) and b) square wave voltammograms (dashed lines) were recorded under same conditions.

5. UV/Vis absorption and fluorescence spectra

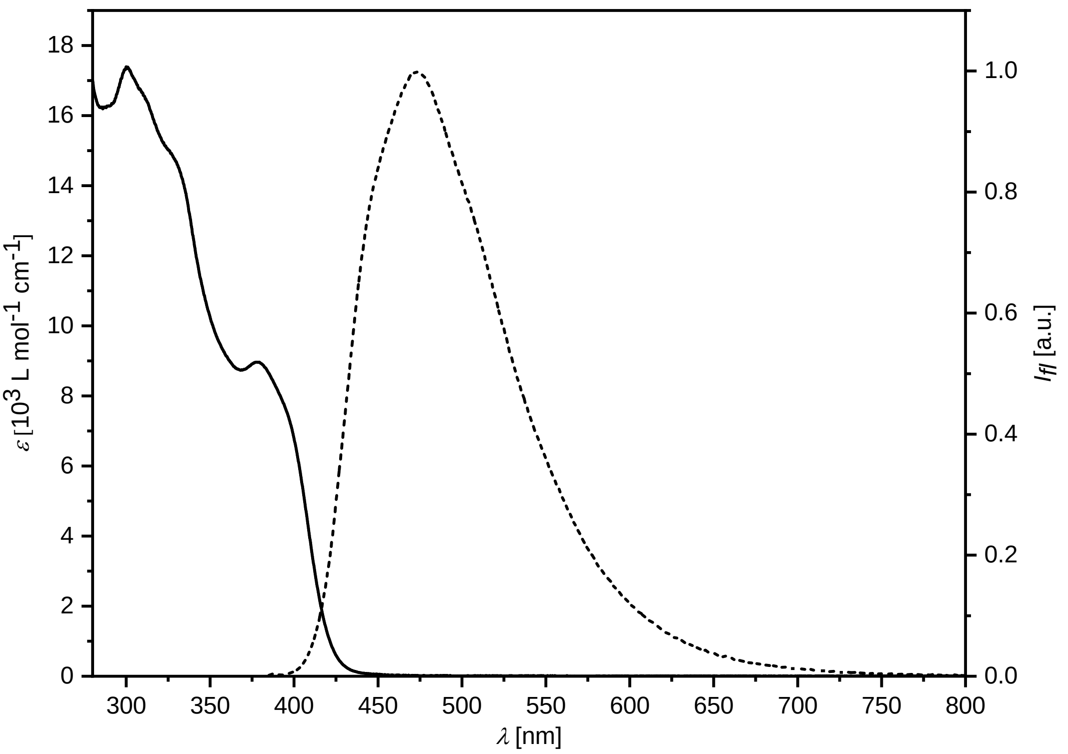

Figure S13. UV/Vis absorption (solid line) and normalized fluorescence (dashed line) spectrum of **1** in CH_2Cl_2 ($c \approx 1\times10^{-5}$ M for UV/Vis, $c \approx 1\times10^{-7}$ M for fluorescence).

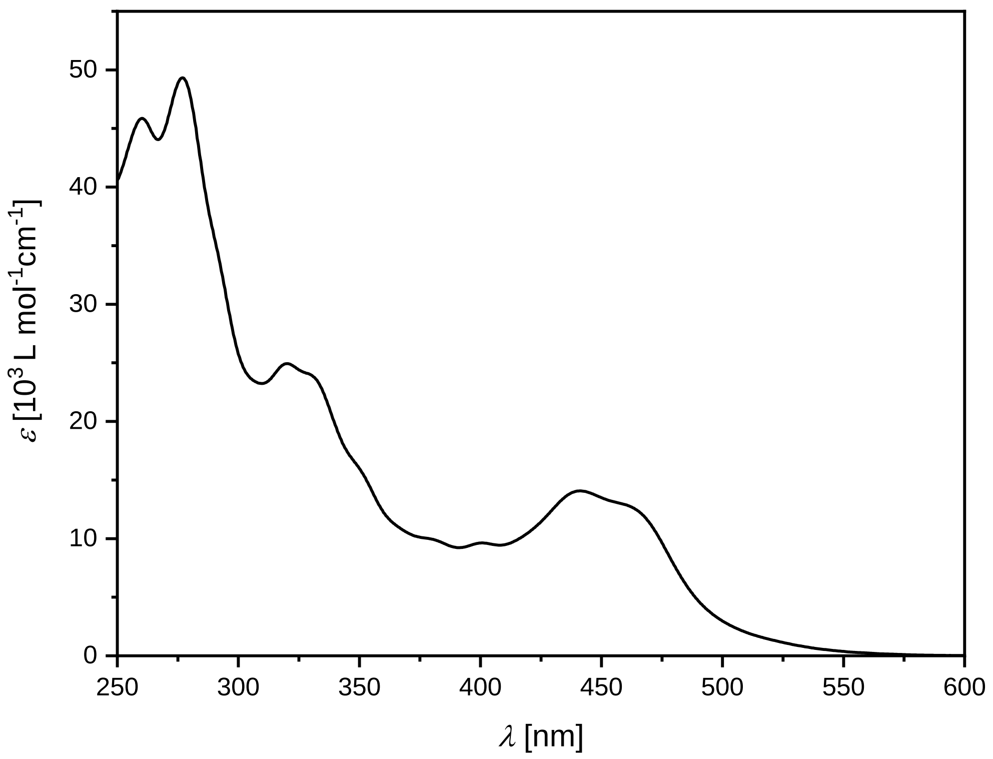

Figure S14. UV/Vis absorption (solid line) spectrum of **2** in CH_2Cl_2 ($c \approx 1\times10^{-5}$ M for UV/Vis). No fluorescence was detected.

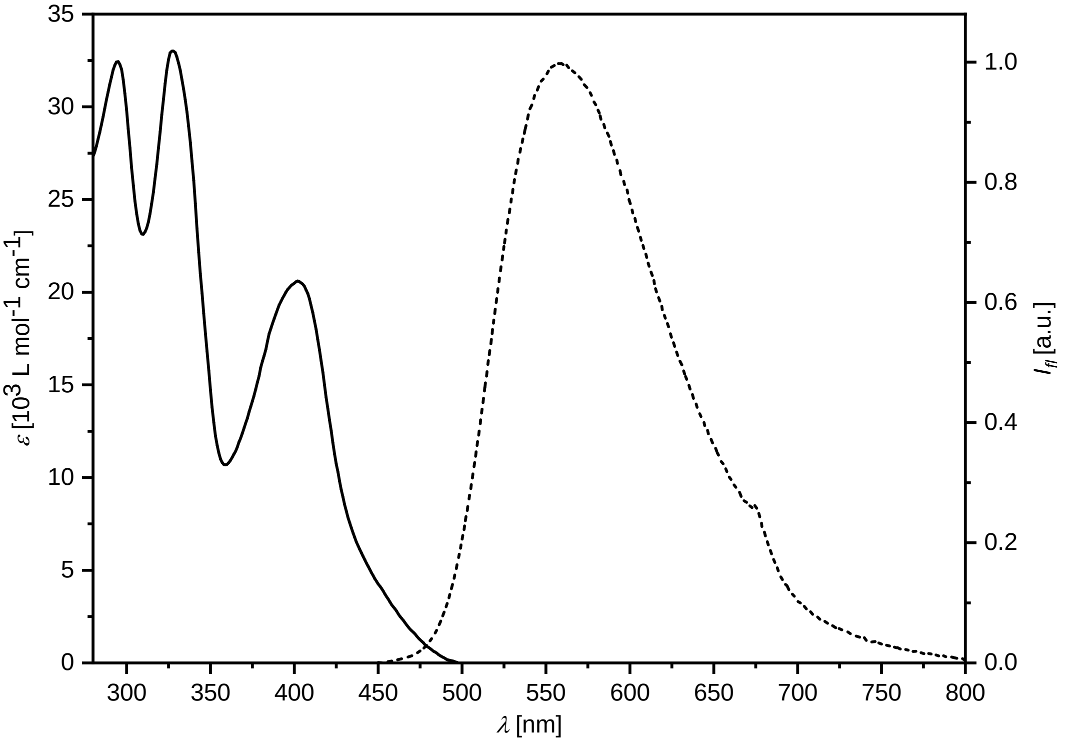

Figure S15. UV/Vis absorption (solid line) and normalized fluorescence (dashed line) spectrum of **3** in CH_2Cl_2 ($c \approx 1\times10^{-5}$ M for UV/Vis, $c \approx 1\times10^{-7}$ M for fluorescence).

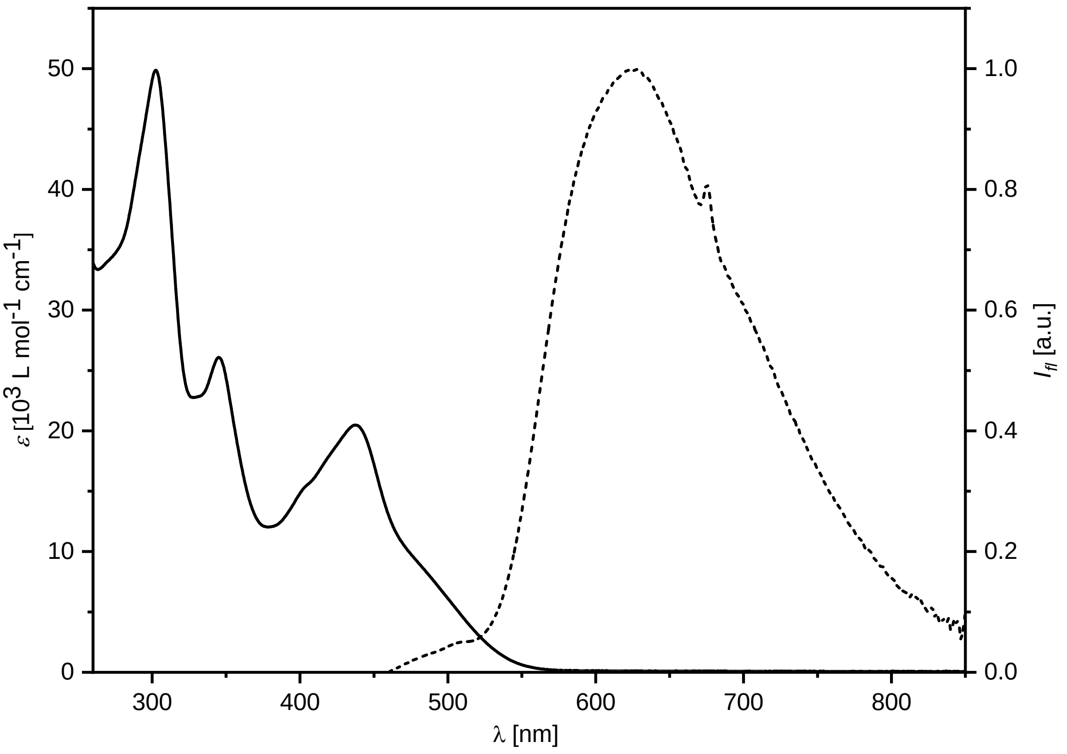

Figure S16. UV/Vis absorption (solid line) and normalized fluorescence (dashed line) spectrum of **4** in CH_2Cl_2 ($c \approx 1\times10^{-5}$ M for UV/Vis, $c \approx 1\times10^{-7}$ M for fluorescence).

6. Crystal structure data

Table S1. Crystallographic data for **1**. CCDC number 2183072.

Empirical formula	$C_{32}H_{20}$	
Formula weight	404.48	
Temperature	120 K	
Wavelength	1.54178 Å	
Crystal system	Orthorhombic	
Space group	$Pna2_1$	
Unit cell dimensions	*a* = 16.1845(5) Å<br>*b* = 7.5542(2) Å<br>*c* = 17.1868(5) Å	α = 90°<br>β = 90°<br>γ = 90°
Volume	2102.27(11) $Å^3$	
Z	4	
Density (calculated)	1.279 g/cm^3	
Absorption coefficient	0.550 mm^{-1}	
F(000)	848	
Theta max	72.371	
Maximum indices	*h* = 19, *k* = 6, *l* = 21	
Reflections collected	33837	
Absorption correction	Semi-empirical from equivalents	
Max. and min. transmission	0.754 and 0.690	

Table S2. Crystallographic data for **3**. CCDC number 2183070.

Empirical formula	$C_{42}H_{26}$	
Formula weight	530.63	
Temperature	100 K	
Wavelength	1.54178 Å	
Crystal system	Triclinic	
Space group	$P\bar{1}$	
Unit cell dimensions	*a* = 5.3137(6) Å<br>*b* = 10.9128(13) Å<br>*c* = 12.1599(15) Å	α = 102.541(5)°<br>β = 97.136(5)°<br>γ = 101.036(5)°
Volume	665.38(14) $Å^3$	
Z	1	
Density (calculated)	1.324 g/cm^3	
Absorption coefficient	0.570 mm^{-1}	
F(000)	278.0	
Theta max	72.665	
Maximum indices	*h* = 6, *k* = 13, *l* = 15	

Reflections collected	2616
Absorption correction	Multi-Scan
Max. and min. transmission	0.754 and 0.617

Table S3. Crystallographic data for **4**. CCDC number 2183071.

Empirical formula	$C_{54}H_{30}$	
Formula weight	678.78	
Temperature	100(2) K	
Wavelength	1.54178 Å	
Crystal system	Triclinic	
Space group	$P\bar{1}$	
Unit cell dimensions	*a* = 4.7724(5) Å	α = 81.412(5)°.
	b = 12.9890(14) Å	β = 83.174(6)°.
	c = 13.5977(15) Å	γ = 84.517(5)°.
Volume	824.94(15) Å3	
Z	1	
Density (calculated)	1.366 g/cm^3	
Absorption coefficient	0.591 mm^{-1}	
F(000)	354	
Crystal size	0.430 × 0.060 × 0.020 mm^3	
Theta range for data collection	3.306 to 80.424°	
Index ranges	$-6 \le h \le 6$, $-16 \le k \le 16$, $-17 \le l \le 17$	
Reflections collected	15763	
Independent reflections	3473 [*R*(int) = 0.0674]	
Completeness to theta = 67.679°	99.3%	
Absorption correction	Semi-empirical from equivalents	
Max. and min. transmission	0.7543 and 0.5296	
Refinement method	Full-matrix least-squares on F^2	
Data / restraints / parameters	3473 / 0 / 244	
Goodness-of-fit on F^2	1.170	
Final *R* indices [*I* > 2sigma(*I*)]	R_1 = 0.1049, wR_2 = 0.3053	
R indices (all data)	R_1 = 0.1155, wR_2 = 0.3116	
Largest diff. peak and hole	0.440, –0.338 e·Å^{-3}	

7. Computational data

7.1. NICS data

Table S4. NICS$_{zz}$ values of pleiadiene and **1**–**4** calculated at the GIAO-B3LYP-6-31+G(d) level of theory. Due to non-planarity of **1**–**4** their values for NICS(1)$_{zz}$ and NICS(–1)$_{zz}$ differ and were averaged to yield NICS(avg.)$_{zz}$.

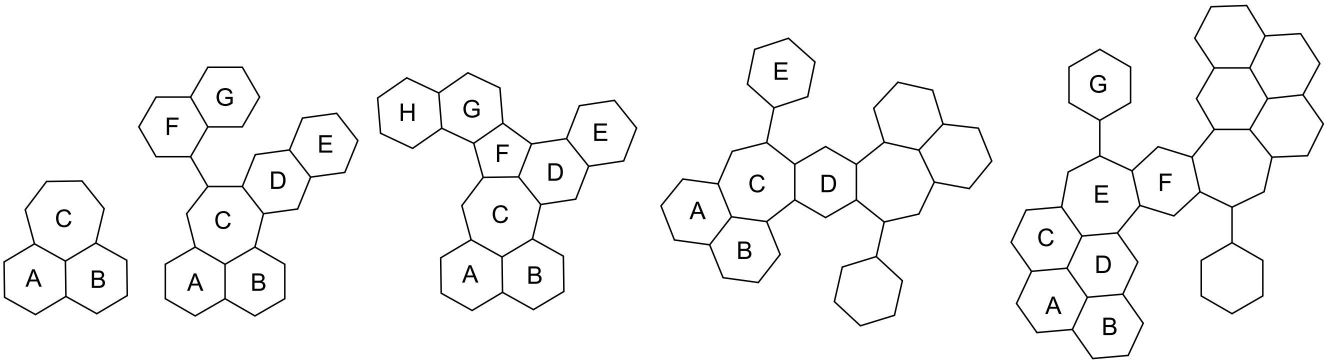

pleiadiene **1** **2** **3** **4**

		A	B	C	D	E	F	G	H
**pleiadiene**	NICS(1)$_{zz}$	-19.6	-19.6	29.8					
**1**	NICS(-1)$_{zz}$	–25.3	–22.0	15.3	–23.4	–25.9	–27.1	–27.7	
	NICS(1)$_{zz}$	–24.1	–25.6	14.1	−23.0	–25.5	–26.9	−28.4	
	NICS(avg.)$_{zz}$	–24.7	–23.8	14.7	–23.2	–25.7	–27.0	−28.0	
**2**	NICS(-1)$_{zz}$	–23.7	–20.8	17.7	–17.6	–23.4	5.5	–22.0	–24.0
	NICS(1)$_{zz}$	–23.9	–23.2	17.3	–17.3	–23.7	8.0	–22.1	–26.4
	NICS(avg.)$_{zz}$	–23.8	–22.0	17.5	–17.5	–23.5	6.8	–22.0	–25.2
**3**	NICS(–1)$_{zz}$	–21.8	–25.9	14.4	−17.1	–28.0			
	NICS(1)$_{zz}$	–26.3	–24.0	13.7	−17.1	–27.2			
	NICS(avg.)$_{zz}$	–24.0	–25.0	14.0	−17.1	–27.6			
**4**	NICS(-1)$_{zz}$	–14.1	–33.0	–14.1	–28.5	17.1	–16.6	–28.7	
	NICS(1)$_{zz}$	–14.7	–33.0	–12.4	–31.4	15.8	–16.6	–27.5	
	NICS(avg.)$_{zz}$	–14.4	–33.0	–13.2	−30.0	16.4	–16.6	–28.1	

7.2 Optimized structures

*Cartesian coordinates of the optimized geometry of **1** at the B3LYP/6-31+G(d) level of theory*

C	2.13487800	-1.74858600	-0.87835500
C	2.88582400	-0.87791900	-0.01193900
C	2.34439100	0.29002100	0.63166200
C	1.17689400	1.07508600	0.12416000
C	-0.07018700	0.53414200	-0.35866400
C	-0.29468400	-0.90072100	-0.62174200
C	0.67390200	-1.80022500	-0.92600300
H	0.30734900	-2.78027500	-1.22724600
C	2.80057300	-2.77505300	-1.54909900
H	2.22078400	-3.43990700	-2.18520500
C	4.17729100	-3.01467800	-1.38581800
H	4.65848500	-3.81356000	-1.94376400
C	4.88132800	-2.28323100	-0.45659300
H	5.92400700	-2.50869600	-0.24542600
C	4.25209900	-1.23738800	0.27211300
C	4.96324000	-0.57227100	1.30677800
H	5.98588400	-0.87655500	1.51712000
C	4.34954500	0.40744800	2.05035300
H	4.86555900	0.87873000	2.88284800
C	3.05776100	0.84464200	1.69346600
H	2.61820400	1.66969900	2.24499100
C	1.32059900	2.45812800	0.14413600
H	2.27808800	2.88266300	0.43037800
C	0.28568100	3.36030800	-0.20384300
C	0.45184000	4.77170600	-0.16475100
H	1.41503900	5.18147000	0.13198500
C	-0.59353100	5.60964100	-0.49239100
H	-0.45881600	6.68771300	-0.45811600
C	-1.84986300	5.07195800	-0.87561900
H	-2.66592100	5.74352000	-1.12977500
C	-2.03638200	3.70678000	-0.92852200
H	-2.99694400	3.29131300	-1.22501700
C	-0.97508600	2.81938100	-0.59762000
C	-1.10279300	1.41292600	-0.66995100
H	-2.04991600	1.00860400	-1.01288900
C	-1.71068400	-1.39235900	-0.72317100
C	-2.55482200	-1.47283400	0.43758500
C	-2.11028600	-1.10487000	1.73890600
H	-1.09425500	-0.74641000	1.86837200
C	-2.94524300	-1.20166600	2.83203600
H	-2.58262700	-0.91490600	3.81585400
C	-4.27204200	-1.67489600	2.68326600
H	-4.92203700	-1.74572800	3.55166200
C	-4.73187800	-2.05027700	1.44007200
H	-5.74688000	-2.42264500	1.31778700
C	-3.89595000	-1.96498800	0.29192900
C	-4.35917800	-2.36662300	-0.99126900
H	-5.37773400	-2.73575800	-1.08995000
C	-3.53091300	-2.29168400	-2.08763800
H	-3.88674400	-2.59884500	-3.06782000
C	-2.20890500	-1.80323500	-1.94834400
H	-1.57203600	-1.73241900	-2.82656200

*Cartesian coordinates of the optimized geometry of **2** at the B3LYP/6-31+G(d) level of theory*

C	1.70697300	-2.07856200	-0.55988600
C	2.79245900	-1.26239600	-0.05608700
C	2.67016600	0.08757800	0.43707800
C	1.53579900	1.01433200	0.15284600
C	0.17028300	0.63716500	0.08675400
C	-0.39033600	-0.71118500	0.00859000
C	0.28447800	-1.82086800	-0.39238600
H	-0.32452400	-2.67823200	-0.66007200
C	2.00041000	-3.32329400	-1.12841100
H	1.17565800	-3.92602100	-1.50052800
C	3.30292300	-3.84292100	-1.19626300
H	3.48003600	-4.80970700	-1.65939900
C	4.33021200	-3.14112400	-0.61044200
H	5.33702000	-3.55094800	-0.58144800
C	4.10058700	-1.87269700	-0.01024500

C	5.17680200	-1.22779700	0.65468400
H	6.14324900	-1.72512800	0.68510400
C	4.98226700	-0.01871300	1.27710400
H	5.78331600	0.45539500	1.83811000
C	3.74001200	0.62938300	1.15121800
H	3.61623000	1.60196600	1.61519100
C	1.80663100	2.36726300	-0.01482400
H	2.83626000	2.70955100	-0.05244200
C	0.79096600	3.34784600	-0.14861700
C	1.13608600	4.71989900	-0.29700000
H	2.18991400	4.98857000	-0.32841700
C	0.16631700	5.69366900	-0.39102800
H	0.44488500	6.73837500	-0.50160700
C	-1.19900200	5.32660800	-0.33698500
H	-1.96698000	6.09331400	-0.40118000
C	-1.56725600	4.00291000	-0.20324600
H	-2.62036900	3.76310400	-0.15946200
C	-0.59577300	2.96713000	-0.11368500
C	-0.87597400	1.56958400	-0.01328300
C	-1.85410000	-0.55897100	0.06759500
C	-2.89198900	-1.53214500	0.19085400
C	-2.68205800	-2.91300800	0.47427000
H	-1.67953400	-3.27487200	0.66850400
C	-3.73923600	-3.79522800	0.56717300
H	-3.54781600	-4.84048000	0.79701500
C	-5.07094800	-3.34857100	0.39092000
H	-5.89461000	-4.05419400	0.46082000
C	-5.31560700	-2.01150600	0.16494200
H	-6.33638900	-1.64869400	0.06379900
C	-4.25146900	-1.07317400	0.07842100
C	-4.51042000	0.31425700	-0.08752800
H	-5.54356900	0.64266100	-0.17673500
C	-3.49320800	1.24350600	-0.10821600
H	-3.75015700	2.28936400	-0.21220000
C	-2.14643100	0.81535600	-0.01682600

*Cartesian coordinates of the optimized geometry of transoid-**3** at the B3LYP/6-31+G(d) level of theory*

C	2.65359166	0.98680957	0.74940899
C	3.90645724	0.63744728	0.13093087
C	4.16752059	-0.61867784	-0.52549124
C	5.37617418	-0.79166922	-1.20078162
H	5.57255770	-1.75099203	-1.67417918
C	6.36840140	0.20492948	-1.23835376
H	7.28715906	0.03461937	-1.79337699
C	6.19616007	1.35387671	-0.50024052
H	6.98670428	2.09809363	-0.43775015
C	4.99430752	1.57798717	0.22520277
C	4.88239225	2.71211356	1.07383400
H	5.72457245	3.39652148	1.14445244
C	3.74369970	2.91305303	1.81685929
H	3.67282328	3.74661417	2.51070899
C	2.63245340	2.06572800	1.63060926
H	1.71517774	2.29058208	2.16550540
C	3.37107941	-1.82854832	-0.31957086
H	3.96581414	-2.73512762	-0.41937072
C	2.07449655	-2.02498845	0.02724516
C	1.05772383	-0.95636413	0.07485699
C	-0.26694342	-1.30567133	-0.24018773
H	-0.47560476	-2.35458354	-0.40485392
C	-1.33712726	-0.40413725	-0.34891360

C	1.63983335	-3.43575295	0.27746467
C	1.92438061	-4.46274549	-0.63816283
H	2.41961416	-4.21871512	-1.57483649
C	1.56179844	-5.78568297	-0.37006050
H	1.78686886	-6.56287320	-1.09648837
C	0.90438201	-6.10687990	0.81990538
H	0.62040688	-7.13503849	1.02928519
C	0.60814775	-5.09316135	1.73715890
H	0.09759720	-5.33144073	2.66702656
C	0.96711892	-3.77231087	1.46608158
H	0.73437349	-2.99117566	2.18536845
C	-2.65359166	-0.98680957	-0.74940899
C	-3.90645724	-0.63744728	-0.13093087
C	-4.16752059	0.61867784	0.52549124
C	-5.37617418	0.79166922	1.20078162
H	-5.57255770	1.75099203	1.67417918
C	-6.36840140	-0.20492948	1.23835376
H	-7.28715906	-0.03461937	1.79337699
C	-6.19616007	-1.35387671	0.50024052
H	-6.98670428	-2.09809363	0.43775015
C	-4.99430752	-1.57798717	-0.22520277
C	-4.88239225	-2.71211356	-1.07383400
H	-5.72457245	-3.39652148	-1.14445244
C	-3.74369970	-2.91305303	-1.81685929
H	-3.67282328	-3.74661417	-2.51070899
C	-2.63245340	-2.06572800	-1.63060926
H	-1.71517774	-2.29058208	-2.16550540
C	-3.37107941	1.82854832	0.31957086
H	-3.96581414	2.73512762	0.41937072
C	-2.07449655	2.02498845	-0.02724516
C	-1.05772383	0.95636413	-0.07485699
C	0.26694342	1.30567133	0.24018773
H	0.47560476	2.35458354	0.40485392
C	1.33712726	0.40413725	0.34891360
C	-1.63983335	3.43575295	-0.27746467
C	-1.92438061	4.46274549	0.63816283
H	-2.41961416	4.21871512	1.57483649
C	-1.56179844	5.78568297	0.37006050
H	-1.78686886	6.56287320	1.09648837
C	-0.90438201	6.10687990	-0.81990538
H	-0.62040688	7.13503849	-1.02928519
C	-0.60814775	5.09316135	-1.73715890
H	-0.09759720	5.33144073	-2.66702656
C	-0.96711892	3.77231087	-1.46608158
H	-0.73437349	2.99117566	-2.18536845

*Cartesian coordinates of the optimized geometry of cisoid-**3** at the B3LYP/6-31+G(d) level of theory*

C	-2.64256100	-1.25865000	0.27898800
C	-3.78564500	-0.91577700	-0.52704400
C	-4.03395200	0.39425800	-1.07434100
C	-5.10835200	0.57173900	-1.94687800
H	-5.29945600	1.56860000	-2.33752800
C	-5.98249900	-0.47553100	-2.28994400
H	-6.79538600	-0.29645100	-2.98872800
C	-5.84409600	-1.69714500	-1.67129500
H	-6.56019800	-2.49556000	-1.85134500
C	-4.78040700	-1.93300600	-0.75825200
C	-4.72016800	-3.16236500	-0.04830700
H	-5.49358700	-3.90596600	-0.22600600
C	-3.72097800	-3.38658900	0.86839400
H	-3.69872500	-4.30212800	1.45368000
C	-2.67976000	-2.44652000	1.00630900
H	-1.85549700	-2.68113100	1.67248300
C	-3.39850900	1.61898900	-0.58929900
H	-4.03617000	2.49237100	-0.71659800

C	-2.21719000	1.85665500	0.03369300
C	-1.13652600	0.86396200	0.18386300
C	0.18573900	1.34152100	0.19816800
H	0.32512800	2.41323200	0.14060800
C	1.33541500	0.53698800	0.22909000
C	-1.97039100	3.25436900	0.51254300
C	-2.11722300	4.35830900	-0.34325000
H	-2.35811800	4.19092800	-1.39012100
C	-1.93717000	5.66192900	0.12818400
H	-2.04867600	6.50089700	-0.55439100
C	-1.60691500	5.88600700	1.46673200
H	-1.46751400	6.89922500	1.83495200
C	-1.45286800	4.79526700	2.32921200
H	-1.20143900	4.95836000	3.37452000
C	-1.62652000	3.49421300	1.85532400
H	-1.50989300	2.65213600	2.53315300
C	2.64261500	1.25874700	0.27880700
C	3.78576500	0.91571800	-0.52705400
C	4.03410700	-0.39438300	-1.07417800
C	5.10870100	-0.57203700	-1.94645400
H	5.29977600	-1.56896500	-2.33695000
C	5.98301400	0.47510200	-2.28944600
H	6.79606000	0.29587000	-2.98800200
C	5.84455000	1.69680100	-1.67096600
H	6.56075600	2.49514200	-1.85092700
C	4.78068100	1.93284300	-0.75818900
C	4.72043200	3.16231700	-0.04844400
H	5.49396000	3.90580600	-0.22612400
C	3.72111700	3.38672500	0.86808100
H	3.69877200	4.30236700	1.45320200
C	2.67985300	2.44672100	1.00599700
H	1.85560900	2.68146700	1.67214600
C	3.39850200	-1.61905200	-0.58921100
H	4.03605200	-2.49251400	-0.71651200
C	2.21714400	-1.85660000	0.03375900
C	1.13653300	-0.86382500	0.18385300
C	-0.18571800	-1.34139800	0.19820800
H	-0.32515600	-2.41310900	0.14070500
C	-1.33538500	-0.53685000	0.22915000
C	1.97018800	-3.25428200	0.51258700
C	1.62609100	-3.49405600	1.85532300
H	1.50943000	-2.65194700	2.53310600
C	1.45227100	-4.79508200	2.32923000
H	1.20067900	-4.95811200	3.37451000
C	1.60635000	-5.88586300	1.46681000
H	1.46682000	-6.89905900	1.83503900
C	1.93680200	-5.66185200	0.12829900
H	2.04832500	-6.50084900	-0.55423800
C	2.11702400	-4.35826200	-0.34315500
H	2.35807600	-4.19093600	-1.39000000

*Cartesian coordinates of the optimized geometry of TS-**3** at the B3LYP/6-31+G(d) level of theory*

C	-2.61661300	-0.90693400	0.92593300
C	-3.88482300	-0.63276100	0.30388300
C	-4.16822500	0.53886600	-0.48686600
C	-5.39609200	0.62979800	-1.14506300
H	-5.60821500	1.52555900	-1.72419000
C	-6.38523200	-0.36443400	-1.04008400
H	-7.32009200	-0.25844300	-1.58408100
C	-6.18502200	-1.42528700	-0.18664600
H	-6.96749000	-2.16259200	-0.02316800
C	-4.96132000	-1.56642100	0.52257600
C	-4.81364800	-2.61560800	1.46925900
H	-5.64795800	-3.29439400	1.62987200
C	-3.64847300	-2.74609900	2.18647600
H	-3.54711600	-3.51641400	2.94648000
C	-2.55393600	-1.90848200	1.89292100
H	-1.61683000	-2.07473300	2.41515000
C	-3.36984700	1.76326200	-0.46997500
H	-3.96293600	2.63978900	-0.72550900
C	-2.07874900	2.02771500	-0.14232500
C	-1.04618300	1.00441000	0.09956400
C	0.29557900	1.35976100	-0.11460800

H	0.43798600	2.39271900	-0.37593400
C	1.42036800	0.51270300	-0.04405700
C	-1.67947700	3.47326600	-0.12579100
C	-1.87819400	4.29577600	-1.24659000
H	-2.27568700	3.86105600	-2.16040900
C	-1.55570100	5.65568900	-1.20627200
H	-1.71236000	6.27214700	-2.08811500
C	-1.02812000	6.21836300	-0.04181900
H	-0.77763000	7.27562400	-0.00843500
C	-0.82076700	5.40937400	1.08081800
H	-0.41530600	5.83772900	1.99428700
C	-1.13818500	4.05096800	1.03709500
H	-0.97750200	3.43014900	1.91509000
C	2.79514300	1.15962300	-0.05642300
C	4.09018300	0.49631000	0.04105700
C	4.33270100	-0.92910700	0.01745300
C	5.63562500	-1.42292100	0.14007000
H	5.77743700	-2.49984800	0.10650300
C	6.75902100	-0.59986800	0.29610500
H	7.74934200	-1.03528000	0.39854300
C	6.57492300	0.75902300	0.29365400
H	7.41969900	1.43676800	0.38896400
C	5.27746900	1.32531800	0.15708200
C	5.19639100	2.73900700	0.13559400
H	6.11242400	3.31836600	0.21937700
C	3.97609500	3.34602800	0.01699800
H	3.88396100	4.42883900	0.00208600
C	2.81623900	2.55986500	-0.05807600
H	1.89707600	3.12157900	-0.08753100
C	3.34918100	-1.97877800	-0.18890700
H	3.81453400	-2.93881800	-0.39942400
C	1.99798800	-2.00884200	-0.16546100
C	1.09374400	-0.87206100	0.07395400
C	-0.23838500	-1.21506300	0.38240700
H	-0.45003200	-2.26484600	0.53175200
C	-1.31749300	-0.33087000	0.47474000
C	1.39188500	-3.35312900	-0.46153700
C	1.67811900	-4.46176000	0.35080700
H	2.29530100	-4.32599200	1.23572200
C	1.17253200	-5.72880600	0.04249300
H	1.40328700	-6.57234400	0.68872300
C	0.37205500	-5.90883400	-1.08739300
H	-0.02166200	-6.89273400	-1.32945400
C	0.07807600	-4.81187000	-1.90479300
H	-0.54104700	-4.94147800	-2.78912200
C	0.57866600	-3.54743900	-1.59224600
H	0.34583700	-2.70092100	-2.23321800

Cartesian coordinates of the optimized geometry of ***4*** *at the B3LYP/6-31+G(d) level of theory*

C	-5.40408434	-2.05651569	-1.81163530
H	-4.85466327	-2.62049205	-2.34314487
C	-6.73400957	-2.35633835	-1.67810557
H	-7.09200264	-3.12663781	-2.10330164
C	-7.56429022	-1.53165878	-0.91744572
H	-8.48389839	-1.75130085	-0.81918822
C	-7.05480862	-0.37852555	-0.29514160
C	-5.67334338	-0.07661795	-0.41920836
C	-7.89180719	0.53355103	0.45129529
H	-8.82255832	0.35830698	0.52926482
C	-7.37743668	1.62036527	1.03361439

H	-7.94826476	2.19518767	1.52900398
C	-5.98636541	1.94108086	0.92787062
C	-5.11298178	1.08169523	0.19719273
C	-5.47297984	3.11325311	1.48217875
H	-6.02717591	3.66461948	2.02199832
C	-4.16736561	3.47040483	1.25051406
H	-3.85775053	4.30817038	1.57517618
C	-3.27046092	2.64148217	0.54899716
C	-3.71716654	1.38057582	0.08124596
C	-2.01296451	3.26914400	0.17906160
H	-2.04079850	4.21839102	0.20561899
C	-0.81689326	2.77602489	-0.19312035
C	-0.46167373	1.33587551	-0.15075214
C	0.87321309	1.01019001	0.11306988
H	1.48807589	1.73055634	0.18600177
C	-1.37995892	0.27436452	-0.27809597
C	-2.85007598	0.37731215	-0.53931099
C	-3.43798950	-0.65759997	-1.22110401
H	-2.88093742	-1.21943335	-1.74749359
C	-4.82991977	-0.93801353	-1.18714923
C	0.23095650	3.75001096	-0.60090164
C	0.99483157	3.55851661	-1.74939021
H	0.87498341	2.76747393	-2.26149144
C	1.92100028	4.49213280	-2.15776344
H	2.42952161	4.33562957	-2.94548682
C	2.12113295	5.65429531	-1.43694311
H	2.74924644	6.30265877	-1.73147926
C	1.38832791	5.86087062	-0.27446356
H	1.52252506	6.65048428	0.23588666
C	0.46571319	4.91976642	0.14094766
H	-0.02019114	5.06684962	0.94419503
C	5.40408434	2.05651569	1.81163530
H	4.85466327	2.62049205	2.34314487
C	6.73400957	2.35633835	1.67810557
H	7.09200264	3.12663781	2.10330164
C	7.56429022	1.53165878	0.91744572
H	8.48389839	1.75130085	0.81918822
C	7.05480862	0.37852555	0.29514160
C	5.67334338	0.07661795	0.41920836
C	7.89180719	-0.53355103	-0.45129529
H	8.82255832	-0.35830698	-0.52926482
C	7.37743668	-1.62036527	-1.03361439
H	7.94826476	-2.19518767	-1.52900398
C	5.98636541	-1.94108086	-0.92787062
C	5.11298178	-1.08169523	-0.19719273
C	5.47297984	-3.11325311	-1.48217875
H	6.02717591	-3.66461948	-2.02199832
C	4.16736561	-3.47040483	-1.25051406
H	3.85775053	-4.30817038	-1.57517618
C	3.27046092	-2.64148217	-0.54899716
C	3.71716654	-1.38057582	-0.08124596
C	2.01296451	-3.26914400	-0.17906160
H	2.04079850	-4.21839102	-0.20561899
C	0.81689326	-2.77602489	0.19312035
C	0.46167373	-1.33587551	0.15075214
C	-0.87321309	-1.01019001	-0.11306988
H	-1.48807589	-1.73055634	-0.18600177
C	1.37995892	-0.27436452	0.27809597
C	2.85007598	-0.37731215	0.53931099
C	3.43798950	0.65759997	1.22110401
H	2.88093742	1.21943335	1.74749359
C	4.82991977	0.93801353	1.18714923
C	-0.23095650	-3.75001096	0.60090164

C	-0.99483157	-3.55851661	1.74939021
H	-0.87498341	-2.76747393	2.26149144
C	-1.92100028	-4.49213280	2.15776344
H	-2.42952161	-4.33562957	2.94548682
C	-2.12113295	-5.65429531	1.43694311
H	-2.74924644	-6.30265877	1.73147926
C	-1.38832791	-5.86087062	0.27446356
H	-1.52252506	-6.65048428	-0.23588666
C	-0.46571319	-4.91976642	-0.14094766
H	0.02019114	-5.06684962	-0.94419503

8. References

[1] M. M. Brahmi, J. Monot, M. Desage-El Murr, D. P. Curran, L. Fensterbank, E. Lacôte, M. Malacria, *J. Org. Chem.* **2010**, *75*, 6983–6985.

[2] J. M. Farrell, C. Mützel, D. Bialas, M. Rudolf, K. Menekse, A. M. Krause, M. Stolte, F. Würthner, *J. Am. Chem. Soc.* **2019**, *141*, 9096–9104.

[3] a) Z. Chen, M. Luo, Y. Wen, G. Luo, L. Liu, *Org. Lett.* **2014**, *16*, 3020–3023; b) K. Park, G. Bae, J. Moon, J. Choe, K. H. Song, S. Lee, *J. Org. Chem.* **2010**, *75*, 6244–6251.

[4] J. Zhang, J. Wang, A. Sandberg, X. Wu, S. Nyström, H. LeVine Iii, P. Konradsson, P. Hammarström, B. Durbeej, M. Lindgren, *ChemPhysChem* **2018**, *19*, 3001–3009.

[5] G. R. Fulmer, A. J. M. Miller, N. H. Sherden, H. E. Gottlieb, A. Nudelman, B. M. Stoltz, J. E. Bercaw, K. I. Goldberg, *Organometallics* **2010**, *29*, 2176–2179.

[6] M. Frisch, G. Trucks, H. Schlegel, G. Scuseria, M. Robb, J. Cheeseman, G. Scalmani, V. Barone, B. Mennucci, G. Petersson, H. Nakatsuji, M. Caricato, X. Li, H. Hratchian, A. Izmaylov, J. Bloino, G. Zheng, J. Sonnenberg, M. Hada, M. Ehara, K. Toyota, R. Fukuda, J. Hasegawa, M. Ishida, T. Nakajima, Y. Honda, O. Kitao, H. Nakai, T. Vreven, J. Montgomery, J. Peralta, F. Ogliaro, M. Bearpark, E. B. J. Heyd, V. S. K. Kudin, J. N. R. Kobayashi, K. Raghavachari, A. Rendell, J. Burant, S. Iyengar, J. Tomasi, M. Cossi, N. Rega, J. Millam, M. Klene, J. Knox, J. Cross, V. Bakken, C. Adamo, J. Jaramillo, R. Gomperts, R. Stratmann, O. Yazyev, A. Austin, R. Cammi, C. Pomelli, J. Ochterski, R. Martin, K. Morokuma, V. Zakrzewski, P. S. G. Voth, J. Dannenberg, S. Dapprich, A. Daniels, O. Farkas, J. Foresman, J. Ortiz, J. Cioslowski, D. Fox, Gaussian 09 Rev A.2, **2009**.

[7] T. Lu, F. Chen, *J. Comput. Chem.* **2012**, *33*, 580–592.
